# Novel therapies for post-stroke cognitive impairment: a systematic review

**DOI:** 10.3389/fneur.2025.1569329

**Published:** 2025-05-27

**Authors:** Katharina Kreiger, Elisabeth Weiss, Felix Fluri

**Affiliations:** ^1^Faculty of Psychology, University of Innsbruck, Innsbruck, Austria; ^2^Kliniken Valens, Rheinburg Klinik, Walzenhausen, Switzerland; ^3^Department of Neurology, University Hospital of Würzburg, Würzburg, Germany

**Keywords:** stroke, post-stroke cognitive impairment (PSCI), rehabilitation, cognitive therapy, brain stimulation

## Abstract

**Background:**

Stroke impacts 15 million people annually, ranking as the second-leading cause of mortality and the third-leading cause of disability globally. Despite advances in acute care, long-term cognitive impairments persist in 30–70% of survivors, impeding rehabilitation and increasing dependency. The existing treatments for post-stroke cognitive impairment (PSCI) show limited efficacy, underscoring the need for more comprehensive approaches. The objective of this systematic review is to evaluate the effectiveness of novel therapeutic interventions for PSCI.

**Methods:**

The present systematic review was conducted in accordance with the PRISMA guidelines and has been registered in PROSPERO (CRD42024621445). A comprehensive search in PubMed and EMBASE identified randomized controlled trials (RCTs) from the past 5 years examining PSCI interventions, with the selection criterion being an assessment of the trials using the Montreal Cognitive Assessment (MoCA). Statistical analyses included pooled mean differences (MD) with 95% confidence intervals (CI), heterogeneity assessment, and subgroup analyses.

**Results:**

Of 755 identified articles, 22 RCTs involving 5,100 participants met the inclusion criteria. The results demonstrated that brain stimulation therapies, particularly transcranial direct current stimulation (tDCS; MD 4.56, 95% CI: 3.19–5.93) and pharmacological interventions (MD 4.00, 95% CI: 3.48–4.52) exhibited significant benefits. Acupuncture showed potential benefits (MD 2.65, 95% CI: 1.07–4.23), albeit with considerable variability. Training approaches yielded mixed outcomes (MD 1.53, 95% CI: −0.09–3.15). Early interventions (within 3 months post-stroke) were the most effective.

**Discussion:**

Brain stimulation, especially tDCS, resulted in consistent cognitive benefits, with early initiation enhancing outcomes. Pharmacotherapy demonstrated robust, generalizable results, while cognitive training showed small but reliable effects. Acupuncture and physical training hold potential but require further standardization.

**Conclusion:**

Effective stroke rehabilitation requires a multimodal, personalized approach integrating brain stimulation, pharmacotherapy, and cognitive training. Early intervention is critical for maximizing neuroplasticity, the effect of later interventions needs further evaluation. Standardization is needed to optimize physical training and alternative medicine.

**Systematic review registration:**

https://www.crd.york.ac.uk/prospero/, identifier CRD42024621445.

## 1 Introduction

Approximately 15 million individuals worldwide are affected by stroke annually, making it the second-leading cause of mortality and the third-leading cause of disability globally (1). Ischemic stroke accounts for a considerable proportion of both global mortality and disability-adjusted life years (DALYs) lost (2, 3). Despite the enhancement of survival rates attributable to advancements in acute stroke care, the long-term impact on cognitive health remains substantial. Cognitive impairments manifest in 30–70% of stroke survivors within the first year, affecting domains such as attention, memory, executive functioning, and visuospatial processing (4). Longitudinal studies indicate that 30% of these individuals experience persistent cognitive deficits years after onset of stroke, with a significant proportion progressing to major neurocognitive disorders (5). Over an average follow-up period of nearly 6 years, 19% of stroke survivors developed dementia, and thus, their dementia risk was 80% higher compared to the general population (6).

Post-stroke cognitive impairment (PSCI) significantly reduces the quality of life of stroke survivors, manifesting as both cognitive and physical disabilities that profoundly affect daily functioning and independence. These impairments not only limit individuals’ ability to perform routine activities but also increase their dependence on caregivers, placing substantial emotional and financial strain on families and healthcare systems (7). Beyond the individual impact, PSCI imposes a considerable societal burden, driven by increased reliance on healthcare resources, prolonged rehabilitation needs, and the economic costs of caregiving. Cognitive deficits result in higher healthcare expenditures due to frequent hospitalizations, extended rehabilitation, and long-term care requirements (8). Moreover, the reduced workforce participation of both stroke survivors and their caregivers further exacerbates these economic challenges (9).

In current rehabilitation settings, PSCI presents substantial challenges: Cognitive impairments hinder patients’ ability to adhere to prescribed therapies, recognize symptoms, and effectively manage their recovery, increasing the risk of recurrent strokes and further cognitive decline (10). Additionally, these deficits limit active participation in rehabilitation programs, reducing the effectiveness of conventional therapeutic interventions and reinforcing long-term dependency on caregivers (11).

Traditional therapy for PSCI primarily focuses on cognitive training targeting specific deficits. However, recent meta-analyses highlight its limitations. For example, attention training provides short-term benefits but fails to produce lasting improvements in cognitive function or quality of life (12). Similarly, interventions targeting visuospatial deficits, such as spatial neglect, lack robust evidence of efficacy (13), while executive function training has not demonstrated statistically significant cognitive improvements (14). A key reason for these shortcomings is growing recognition that stroke is a network disorder rather than a focal lesion, leading to widespread disruptions in brain organization and function. Cognitive impairments result not only from localized damage but also from disturbances in large-scale brain network connectivity, as demonstrated by neuroimaging studies (15). This shift in perspective suggests that traditional training approaches, which focus on specific cognitive domains, may be insufficient to address the complex neural reorganization required for recovery. Importantly, observed cognitive improvements may result more from spontaneous recovery and neuroplasticity than from the direct effects of cognitive training (16, 17).

Given the high prevalence and significant impact of PSCI, there is an urgent need to identify the most effective therapeutic strategies to enhance cognitive recovery. While various interventions have been explored, their comparative effectiveness remains unclear. The objective of this systematic review is to critically evaluate the efficacy of novel therapeutic interventions for PSCI, including brain stimulation techniques, pharmacological treatments, and training-based approaches. By synthesizing current evidence, this review aims to provide insights into which treatments offer the most substantial cognitive benefits, ultimately guiding future rehabilitation practices and improving patient outcomes.

## 2 Methods

### 2.1 Protocol and registration

The present systematic review was conducted in accordance with the guidelines set out in the Preferred Reporting Items for Systematic Reviews and Meta-Analyses (PRISMA) statement. The protocol was published in the International Prospective Register of Systematic Reviews (PROSPERO) on December 2nd, 2024 (CRD42024621445).

### 2.2 Literature search strategy

A systematic search was conducted in the databases PubMed and EMBASE (January 2020 to December 2024) in December 2024. For the search we used a combination of keywords and Medical Subject Headings (MeSH) related to stroke (e.g., “stroke,” “cerebrovascular accident,” “cerebral ischemia”), cognitive impairment (e.g., “dysfunction,” “deficit,” “attention,” “memory”), and rehabilitation (e.g., “therapy,” “training”). It should be noted that the final selection comprised articles written in English. The search terms and full protocol are available in our PROSPERO registration (ID: CRD42024621445).

### 2.3 Selection criteria

The following inclusion criteria were applied: (1) randomized controlled trials (RCTs) published in the last 5 years (i.e., January 2020 to December 2024) that evaluated interventions for post-stroke cognitive impairment; (2) male and female individuals who had experienced a stroke within the last 6 months before intervention; (3) stroke-related cognitive impairments which have been proven using the Montral Cognitive Assessment (MoCA) (18); (4) any therapeutic interventions specifically targeting cognitive impairment. Excluded were studies using other tests as the MoCA to determine cognitive deficits. Comparator interventions were either placebo or standard care. The primary outcome measure was any improvement in MoCA scores of the experimental group compared to the control group. The overarching research question guiding this systematic review was: What is the effectiveness of novel therapeutic interventions compared to standard care in improving cognitive outcomes in individuals with post-stroke cognitive impairment?

### 2.4 Outcome measures

The cognitive functions of participants were evaluated before and after intervention using the MoCA. The MoCA is a cognitive screening tool that requires the patient to complete 11 tasks assessing attention/concentration, executive functions, memory, language, visuoconstructional skills, conceptual thinking, calculations, and orientation. The test takes approximately 10 min. The total possible score is 30 points, scores ≥ 26 points are considered normal. The primary outcome of the study was the between-group difference of scores in the MoCA before and after intervention.

### 2.5 Data extraction

The characteristics of the study (authors, publication year, country, sample size), the participating patients (age, sex, stroke type, time of stroke), the intervention (treatment, frequency, duration, type of comparator), and outcome data were extracted from the full text studies. Screening and data extraction were performed independently by two reviewers. In case of disagreement, a consensus discussion was held to resolve any discrepancies.

### 2.6 Quality assessment

We used the Cochrane Risk of Bias Tool 2 (RoB 2) (19) to assess the quality and risk of bias. The evaluation was based on the following domains: (1) randomization process; (2) deviations from intended interventions; (3) missing outcome data; (4) measurement of the outcome; (5) selection of the reported results. The application of these domains resulted in an estimation of the overall risk of bias.

### 2.7 Statistical analysis

The statistical comparisons were performed in Review Manager by The Cochrane Collaboration (20). The change of MoCA scores were entered as continuous variables with means and standard deviations (SD). The mean difference (MD) and 95% confidence intervals (CI) were calculated as pooled estimate. Between-study heterogeneity was assessed using the I^2^ statistic and chi-square test. I^2^ values of 0–40% was interpreted as low heterogeneity, 40–75% as moderate, and >75% as substantial heterogeneity. When heterogeneity was significant (I^2^ > 50%), random models were used, and subgroup analyses were performed to explore potential sources.

### 2.8 Subgroup analysis

The selected studies were categorized by intervention type (brain stimulation, medication, alternative medicine approaches and cognitive and/or physical training) due to the variety of novel therapeutic approaches included in the review. Further subgroup analyses were pre-specified to examine specific intervention types in greater detail. Additionally, sensitivity analyses were conducted to assess the robustness of the results and to evaluate the impact of potential sources of bias on the findings.

## 3 Results

### 3.1 Study selection

**Figure 1:**
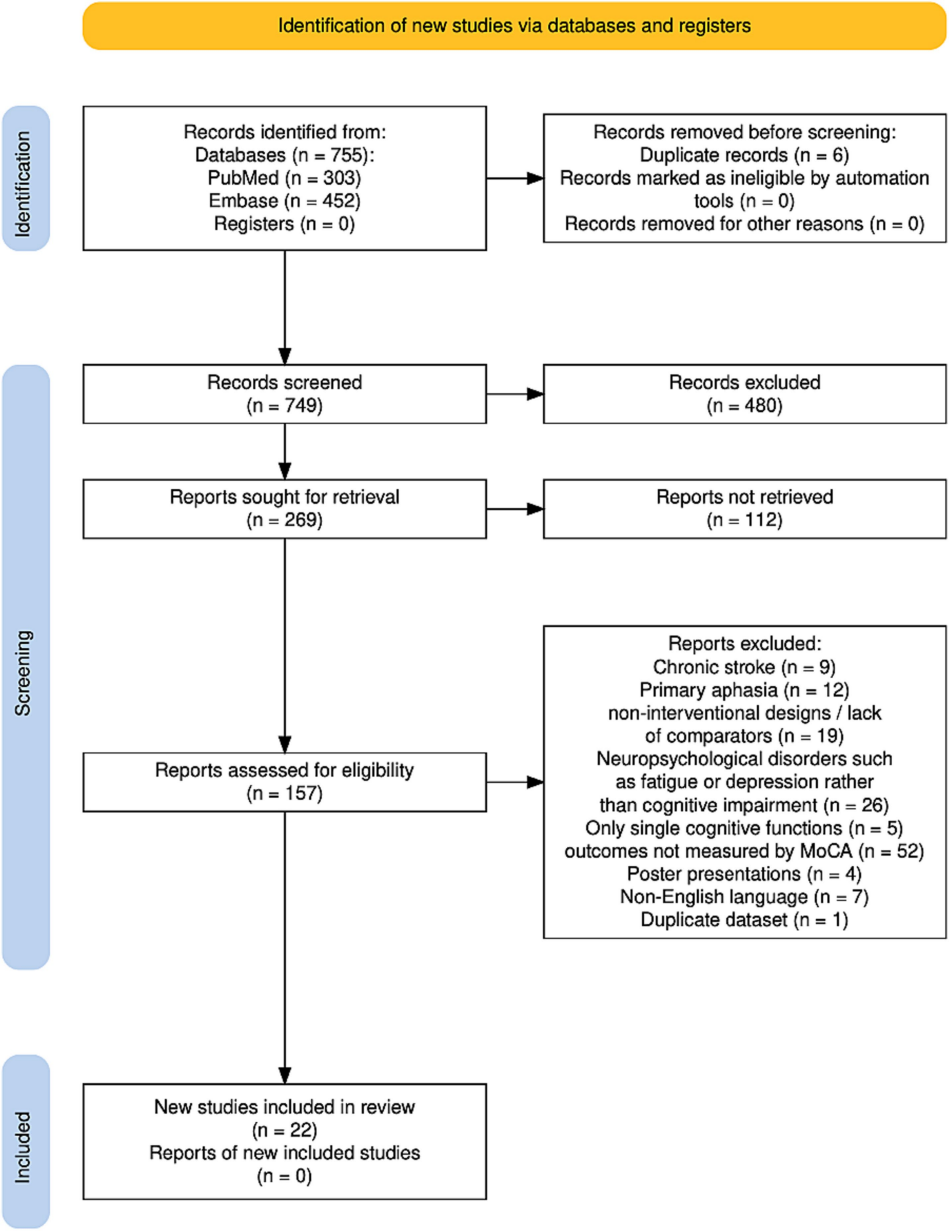
Study flow diagram.

The results of the search are summarized in Figure 1. The study flow diagram was created using software by Haddaway et al. (21). A total of 755 studies were identified, 303 studies from PubMed and 452 studies from Embase. After removing 6 duplicates, the studies were screened based on title and abstract and 269 studies were selected for further consideration. Out of them, full text of 112 studies could not be retrieved. Another 135 trials have been excluded for the following reasons: the focus of these studies was on chronic stroke (9 studies), mainly on aphasia or speech-related disorders (12 studies), on neuropsychological disorders such as fatigue or depression rather than cognitive impairment (26 studies), trials providing non-interventional designs or lack of comparators (19 studies), and studies targeting only single cognitive functions (5 studies), assessment of PSCI was performed by another test than MoCA (52 studies). Four articles were excluded because they were poster presentations, seven articles were not available in English, and one study was excluded because it used the same dataset as another study. Finally, 22 studies were identified fulfilling all inclusion criteria.

### 3.2 Study characteristics

All included studies are summarized in Table 1. A total of 22 studies encompassing 5,100 stroke survivors fulfilled the inclusion criteria and were included in the meta-analysis. The mean age of the participants was 62.64 years, with 2,195 being male. Patients with either an ischemic stroke or a hemorrhagic stroke were included in the analysis. It is noteworthy that each intervention was administered in conjunction with conventional cognitive rehabilitation programs. More than 90% of the trials were conducted in China, whereas no studies were performed in Europe or America.

**Table 1:** Study characteristics.

Author (Year)	Treatments / Comparator	Duration	Frequency
Ai et al. (54)	transcranial direct current stimulation	2 weeks	5 x / week for 2 weeks
	sham tDCS		
Chen et al. (29)	Cluster needling of scalp acupuncture	≤ 6 months	2 x / day for 4 weeks
	Drug treatment only		
Chen et al. (55)	Transcranial direct current stimulation and computer training	30 days	5 x / week for 3 weeks
	cognitive training		
Feng et al. (31)	Remote limb conditioning	≤ 14 days	1 x / day for 6 months
	standard treatment		
He et al. (56)	Eye movement training	≤ 6 months	6 x / week for 6 weeks
	Routine treatment		
Hu et al. (22)	rTMS + Galantamine + cognitive training	≤ 6 months	5 x / week for 4 weeks
	rTMS + cognitive training;		
	Galantamine + cognitive training		
Jiang et al. (28)	Butylphthalide + Oxiracetam + standard rehabilitation	≤ 14 days	1 x / days IV for 2 weeks, 1 x / days oral for 12 weeks
	Oxiracetam + routine treatment		
Li et al. (57)	rTMS + cognitive training	≤ 3 months	5 x / week for 3 weeks
	sham stimulation + cognitive training		
Li et al. (58)	rTMS + cognitive training	14 days	5 x / week for 4 weeks
	sham rTMS + cognitive training		
Li et al. (32)	Finger exercise training	≤ 6 months	2 x / day for 3 months
	routine nursing		
Lu et al. (26)	Percutaneous mastoid electrical stimulation + antidepressants	≤ 14 days	1 x / day for 6 months
	sham stimulation + antidepressants		
Qurat-ul-ain et al. (23)	M1 tDCS	2–6 weeks	5 x /week for 3 days
	cerebellar tDCS		
	sham stimulation		
Shang et al. (59)	Handgrip training	≤ 10 days	3 x / week for 12 weeks
	standard rehabiltation		
Sun et al. (60)	Cognitive_motor Dual Task training	approx. 3 months	5 x / week for 4 weeks
	Cognitive Training alone		
Tian et al. (27)	Ginkgo diterpene lactone meglumine	≤ 2 days	1 x / day for 2 weeks
	Placebo		
Wang et al. (61)	Rehabilitation training	≤ 3–6 days	1 x / day for 8 weeks
	drug therapy only		
Wilson et al. (62)	Virtual Reality training	3–4 months	3–4 x / week for 8 weeks
	control training		
Yang et al. (63)	Acupuncture based on the four seas theory	≤ 6 months	3 x / week for 8 weeks
	Conventional basic rehabilitation training		
Yin et al. (25)	rTMS + cognitive training	1–6 months	5 x / week for 4 weeks
	cognitive training + sham stimulation		
Yu et al. (64)	Intermittent theata burst stimulation + cognitive training	≤ 6 months	5 x / week for 4 weeks
	Cognitive Training alone		
Zhang et al. (24)	tDCS + motor-cognitive training	1–3 months	5 x / week for 4 weeks
	tDCS alone;		
	motor-cognitive training alone		
Zhang et al. (30)	Interactive dynamic scalp acupuncture	15–180 days	6 x / week for 8 weeks
	combination therapy		

tDCS, Transcranial Direct Current Stimulation; rTMS, Repetitive Transcranial Magnetic Stimulation; IV, Intravenous; M1, Primary Motor Cortex.

### 3.3 Risk of bias within the studies

The risk of bias was evaluated using the Cochrane Risk of Bias 2 and is summarized in Supplementary Table S2 (RoB 2 tool) (19), with the studies evaluated on the following five domains: (D1) randomization process, (D2) deviations from intended interventions, (D3) missing outcome data, (D4) measurement of the outcome, and (D5) selection of the reported results. Overall, the studies demonstrated a high methodological quality, with an overall low risk of bias observed in all assessed domains. However, there is some uncertainty about the selection of the reported results, as pre-specified protocols and outcomes were not identified. In summary, overall risk of bias assessment ranged between “no concerns” and “some concerns.” Notably, no studies were excluded following this assessment.

### 3.4 Synthesis of results

All patients were assessed using the MoCA test before and after the intervention. First, the studies were grouped based on the type of intervention.

1) Brain stimulation interventions were identified in 10 studies, including 352 patients in the experimental groups and 331 in the control groups;
2) Alternative medicine approaches using acupuncture were classified in 3 studies, with 263 patients in the experimental groups and 285 in the control groups;
3) Pharmacotherapeutic interventions were reported in 2 studies, involving 1,628 patients receiving cognitive-enhancing medication and 1,615 receiving placebo;
4) Training-based interventions were used in 7 studies, with 288 patients in the experimental groups and 338 in the control groups.

All control groups received standard rehabilitation. Some studies [e.g., (22–24)] included more than one comparator group. In order to enhance the comparability of the studies, only comparator groups that were most similar to those in other studies were included in the analysis. Specifically, placebo groups (e.g., sham interventions or standalone training) were used for this purpose.

Second, further analyses were performed to divide the main intervention categories (brain stimulation, pharmacotherapy, alternative medicine, and training) into subgroups based on the type of intervention, comparators, and the onset time of the intervention.

### 3.5 Brain stimulation methods

**Figure 2:**
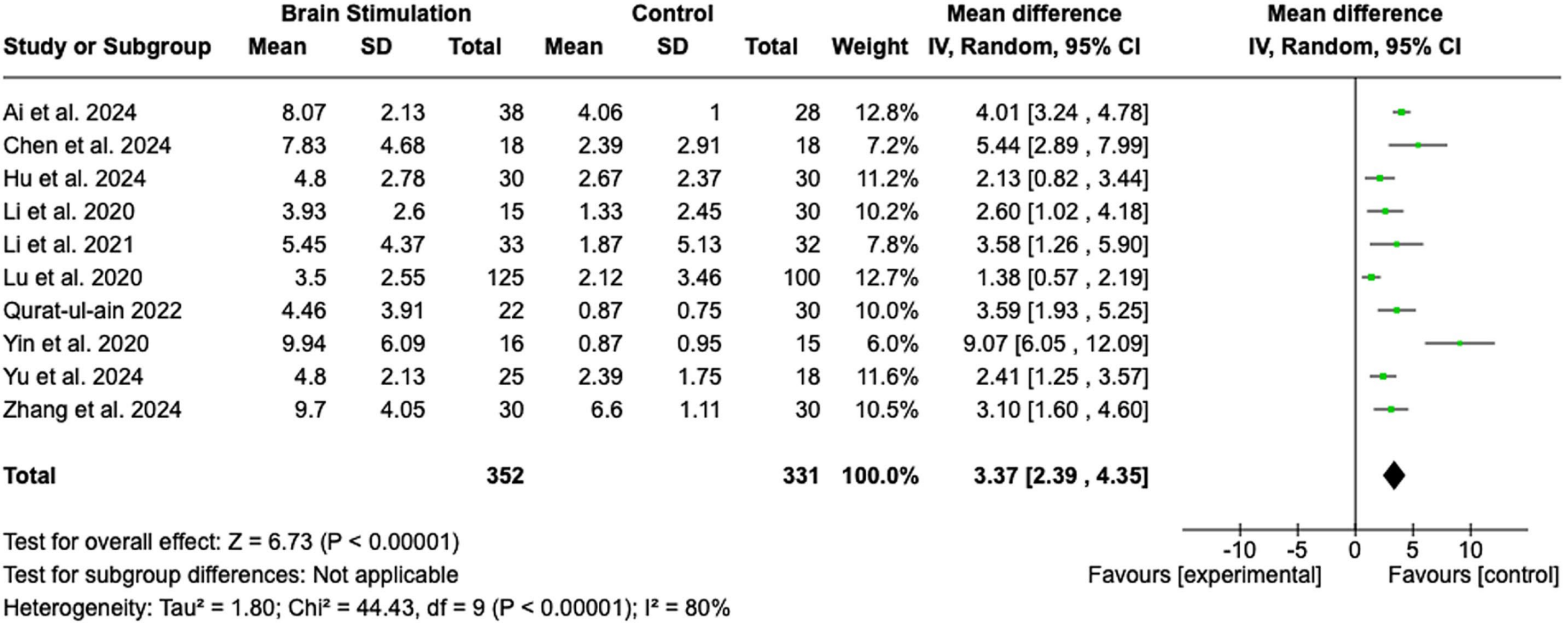
Brain stimulation. SD, Standard Deviation; IV, Inverse Variance; CI, Confidence Interval; df, Degrees of Freedom.

The pooled analysis is shown in Figure 2. The analysis revealed a statistically significant MD favoring brain stimulation therapies over control interventions, with a MD of 3.37 (95% CI: 2.39 to 4.35; Z = 6.73; *p* < 0.00001). The heterogeneity among the included studies was substantial, as indicated by an I^2^ of 80% (Tau^2^ = 1.80; Chi^2^ = 44.43, df = 9; *p* < 0.00001), suggesting considerable variability in study results. Despite this, the random effects model was used to account for the variability between studies, providing a robust estimate of the overall effect. The individual studies contributed weighted mean differences ranging from 1.38 (95% CI: 0.57 to 2.19) to 9.07 (95% CI: 6.05 to 12.09). Notably, the study by Yin et al. (25) reported the largest effect size (MD = 9.07, 95% CI: 6.05 to 12.09), while the study by Lu et al. (26) reported the smallest effect size (MD = 1.38, 95% CI: 0.57 to 2.19). This range indicates variation in the magnitude of benefit between studies, which was further explored in the next step using subgroup analyses.

### 3.6 Subgroup by comparator type (sham stimulation vs. no stimulation)

**Figure 3:**
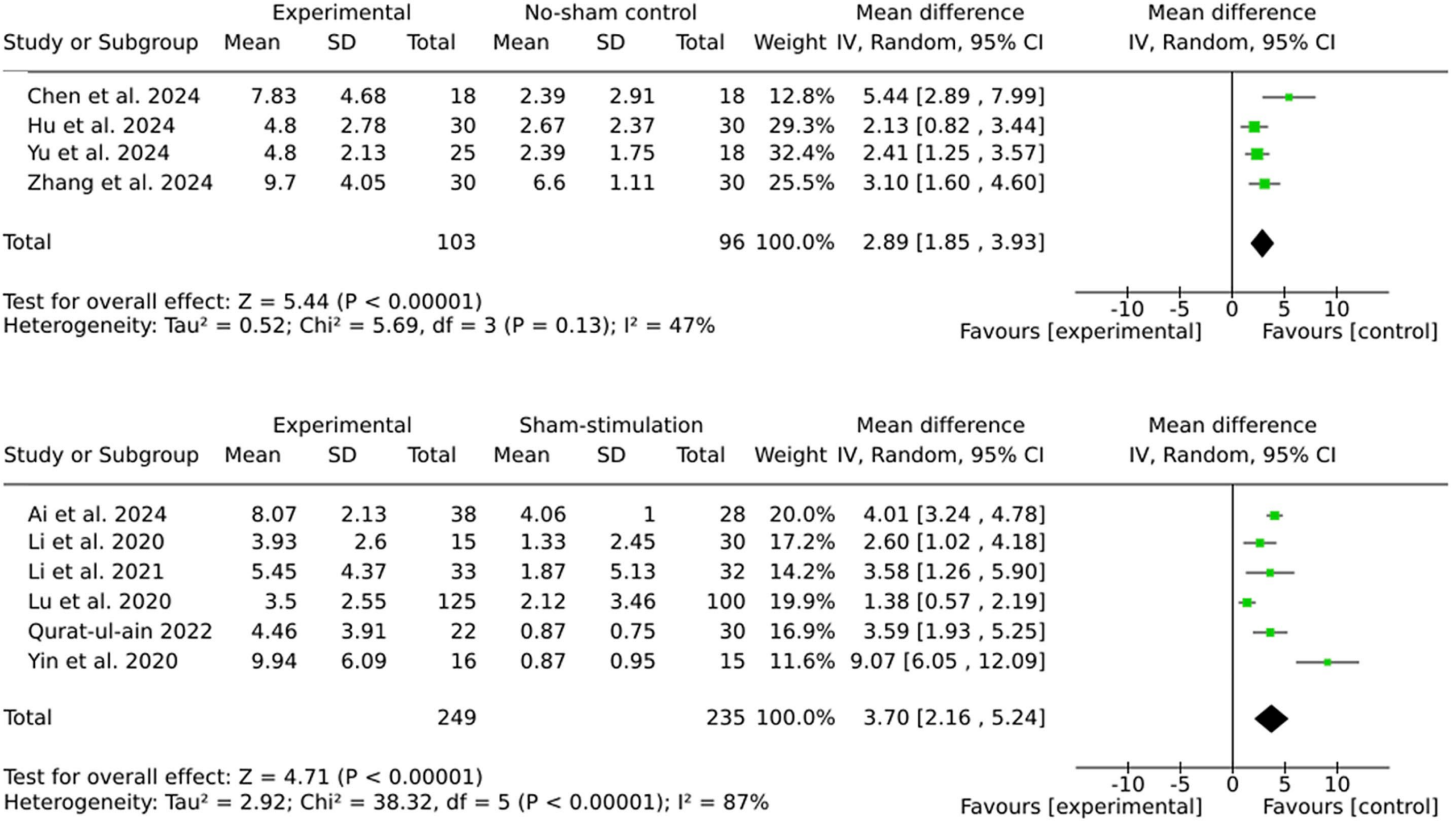
Subgroup by comparator type.

The pooled effect sizes for both subgroups indicate significant benefits of brain stimulation therapies (Figure 3). However, the larger effect size observed in the sham stimulation subgroup (MD = 3.70) compared to the no stimulation subgroup (MD = 2.89) suggests the potential presence of placebo effects in the sham-controlled trials. The subgroup analyses revealed that the sham stimulation subgroup exhibited significantly higher heterogeneity (I^2^ = 87%) in comparison to the no stimulation subgroup (I^2^ = 47%).

### 3.7 Subgroup by onset (start of intervention within 3 or 6 months after stroke)

**Figure 4:**
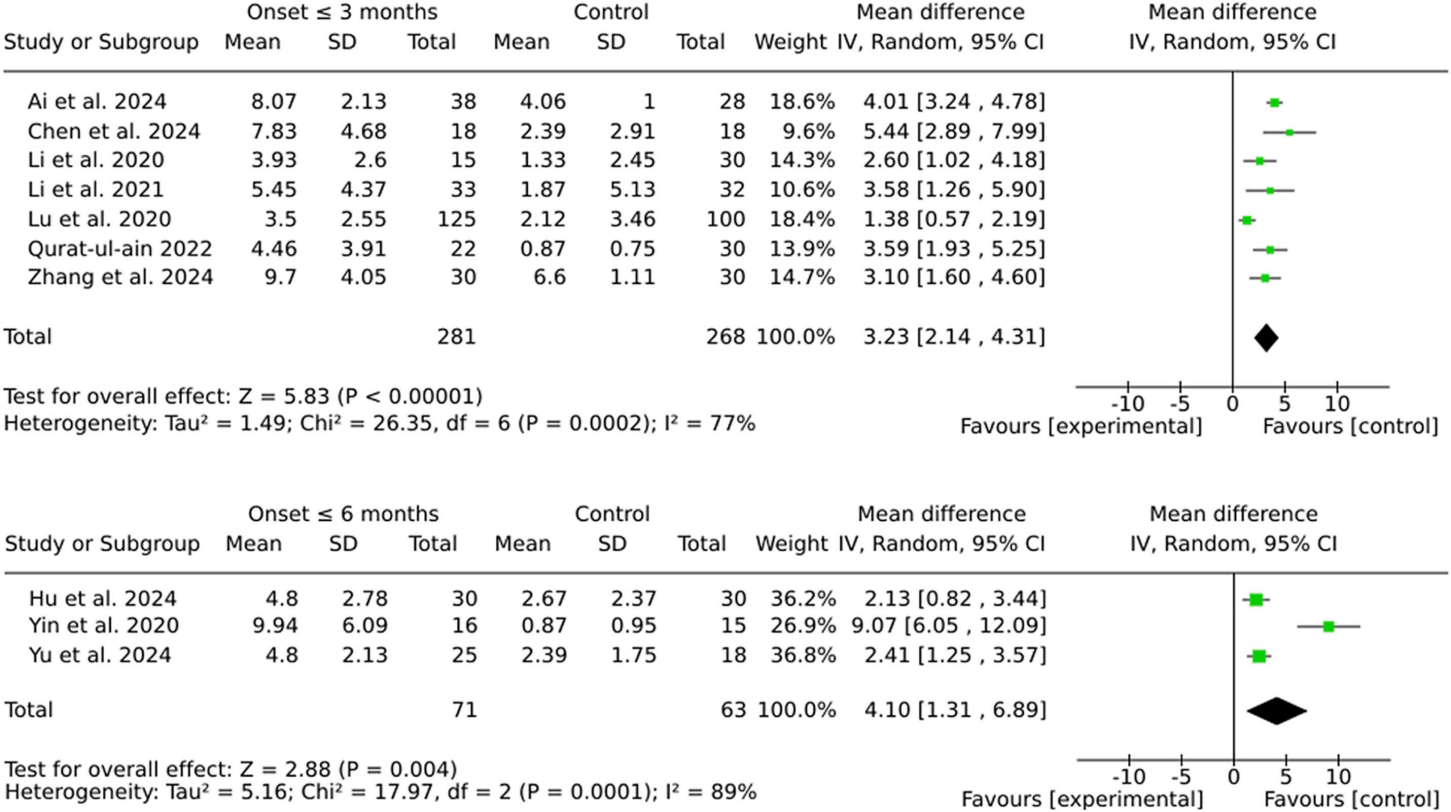
Subgroup by onset.

The pooled MD was marginally higher for the 6-month group (4.10) than the 3-month group (3.23). However, this discrepancy is likely attributable to the greater variability observed in the 6-month group, as indicated by the substantial heterogeneity (I^2^ = 89%). In contrast, the 3-month group demonstrated more consistent effect sizes and reduced variability, thereby ensuring enhanced reliability of the results. Furthermore, individual effect sizes in the 3-month group were consistently higher across a larger number of studies (see Figure 4).

### 3.8 Subgroup rTMS vs. tDCS

**Figure 5:**
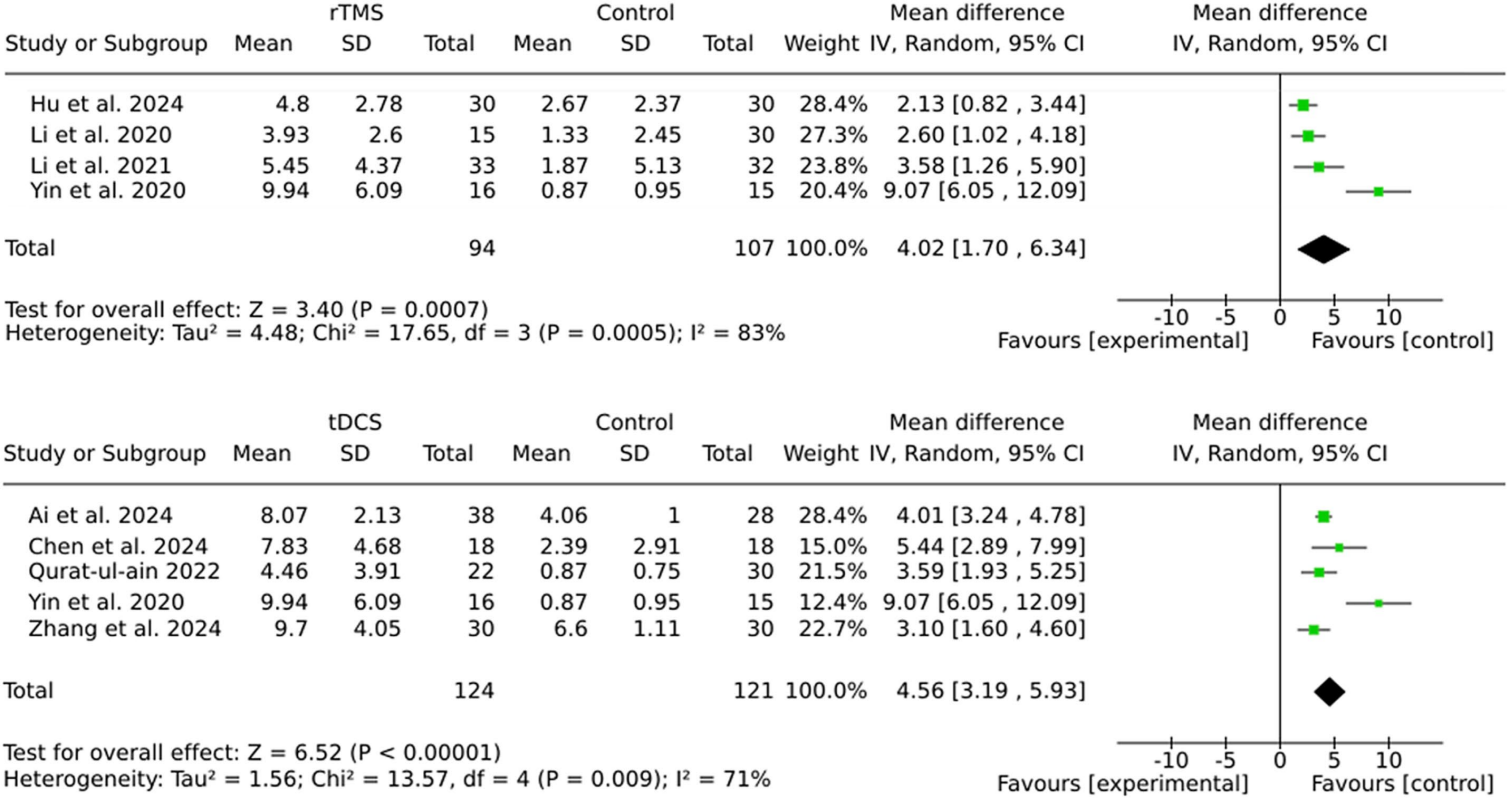
Subgroup by stimulation method.

The findings of this study demonstrate that both rTMS and tDCS are effective in enhancing outcomes in stroke rehabilitation, with tDCS showing a slightly larger pooled effect size (MD = 4.56) compared to rTMS (MD = 4.02). Moreover, the heterogeneity was lower in the tDCS subgroup (I^2^ = 71%) than in the rTMS subgroup (I^2^ = 83%), indicating more consistent results with tDCS (see Figure 5). The variability in outcomes associated with rTMS may be attributable to variations in stimulation parameters (e.g., frequency, intensity) or patient characteristics across studies.

### 3.9 Pharmacotherapeutic interventions

**Figure 6:**
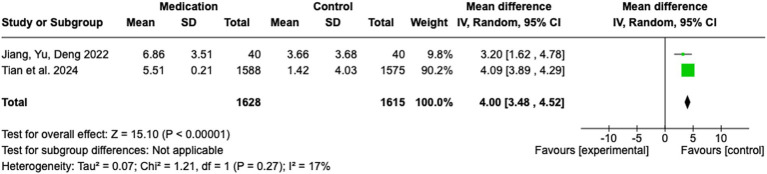
Pharmacotherapeutic interventions.

In this meta-analysis, two studies were included to evaluate the effect of medication-based interventions on stroke rehabilitation outcomes. The pooled analysis yielded a significant MD of 4.00 (95% CI: 3.48 to 4.52; Z = 15.10; *p* < 0.00001), indicating a strong preference for the medication group over the control group (see Figure 6). The heterogeneity across studies was minimal, with an I^2^ of 17% (Tau^2^ = 0.07; Chi^2^ = 1.21, df = 1; *p* = 0.27), indicating negligible variability in effect sizes. This consistency serves to reinforce the robustness of the pooled estimate. The study by Tian et al. (27) contributed most of the weight (90.2%) due to its substantial sample size (n = 1,588 / 1,575). The effect size in this study was 4.09 (95% CI: 3.89 to 4.29). In contrast, the smaller study by Jiang, Yu, and Deng (28) exhibited a MD of 3.20 (95% CI: 1.62 to 4.78), accounting for 9.8% of the total weight.

### 3.10 Alternative medicine

**Figure 7:**
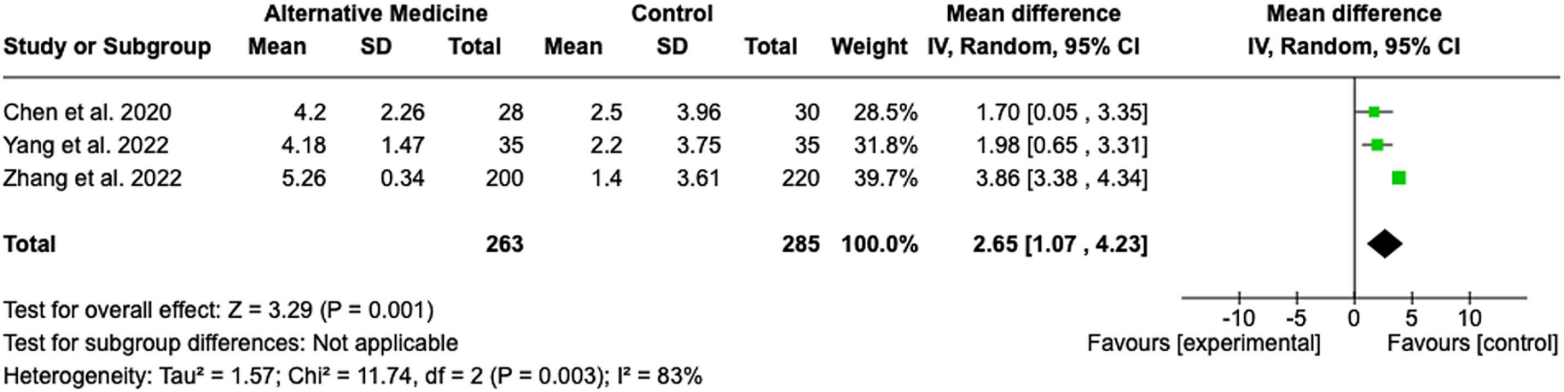
Alternative medicine approaches.

A total of three studies were included in the evaluation of the effectiveness of acupuncture in improving cognitive deficits in stroke rehabilitation (see Figure 7). The pooled MD was 2.65 (95% CI: 1.07 to 4.23; Z = 3.29; *p* = 0.001), indicating a statistically significant benefit of acupuncture compared to control conditions. The heterogeneity among the included studies was substantial, with an I^2^ of 83% (Tau^2^ = 1.57; Chi^2^ = 11.74, df = 2; *p* = 0.003). Despite the high heterogeneity, the studies were similar in terms of onset time, patient characteristics, and intervention protocols. This suggests that factors such as unmeasured confounders or variations in implementation may contribute to the observed variability. Individual study effect sizes ranged from MD 1.70 (95% CI: 0.05 to 3.35) (29) to MD 3.86 (95% CI: 3.38 to 4.34) (30).

### 3.11 Training methods

**Figure 8:**
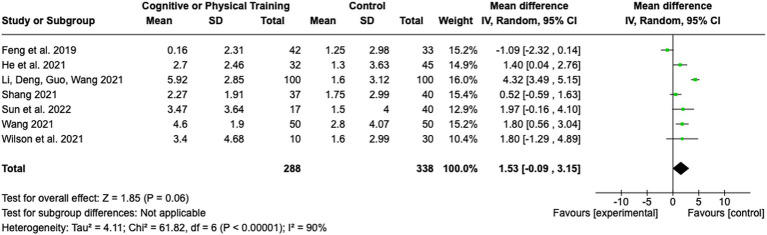
Training methods.

Seven studies assessed the impact of cognitive or physical training interventions on stroke rehabilitation outcomes (see Figure 8). The pooled MD was 1.53 (95% CI: −0.09 to 3.15; Z = 1.85; *p* = 0.06), indicating a trend toward benefit from training interventions, though the result did not reach statistical significance. The heterogeneity among studies was found to be substantial, as indicated by an I^2^ of 90% (Tau^2^ = 4.11; Chi^2^ = 61.82, df = 6; *p* < 0.00001), reflecting considerable variability among the included studies. The effect sizes of the individual studies ranged from a MD of −1.09 (95% CI: −2.32 to 0.14) (31) to an MD of 4.32 (95% CI: 3.49 to 5.15) (32). This wide range of effect sizes may stem from differences in the type of training (physical vs. cognitive), duration and intensity of the interventions, or patient characteristics.

### 3.12 Subgroup by intervention type (cognitive training or physical training)

**Figure 9:**
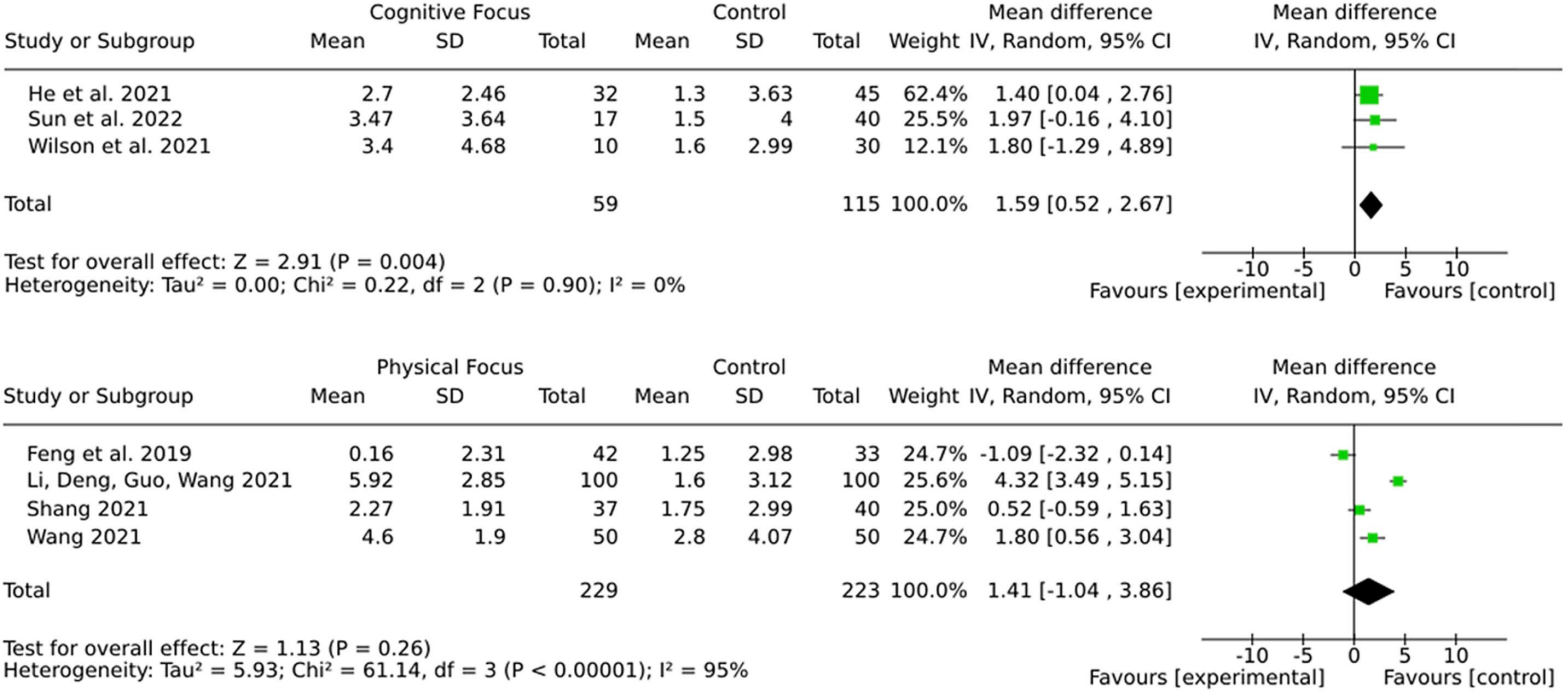
Subgroup by intervention type.

The results (Figure 9) demonstrate a more robust and consistent effect for cognitive training in comparison to physical training regarding the improvement of cognitive outcomes in stroke patients. While cognitive training demonstrated a statistically significant pooled effect and no heterogeneity, physical training showed a non-significant pooled effect with substantial variability. These findings suggest that cognitive training may be more reliable and effective for targeting cognitive deficits after stroke.

### 3.13 Subgroup by onset (3 months or 6 months)

**Figure 10:**
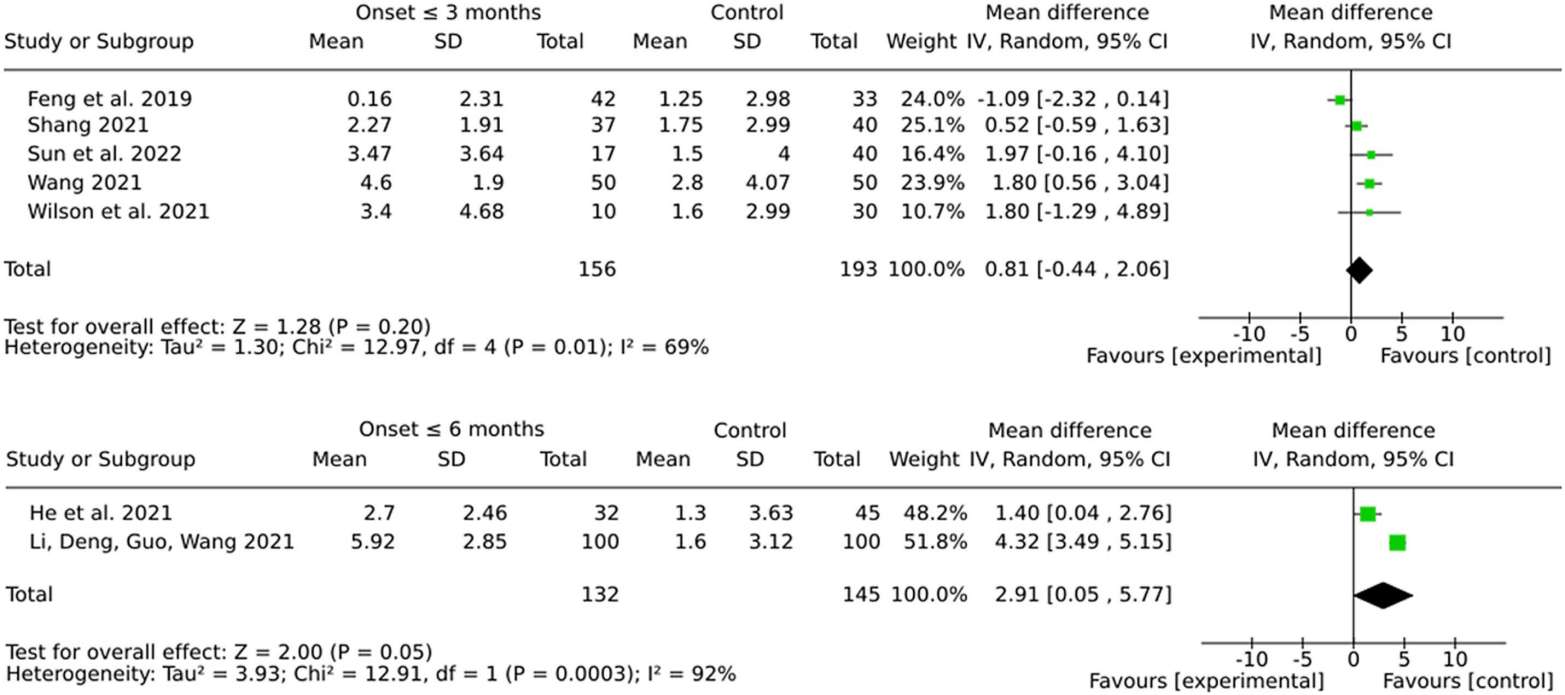
Subgroup by onset.

Interventions initiated within 6 months post-stroke showed a more pronounced and statistically significant pooled effect in comparison to those initiated within 3 months (see Figure 10). However, the considerable heterogeneity observed in both subgroups, particularly in the 6-month group, suggests that variability in protocols, patient populations, or other factors may potentially influence these results. The more modest pooled effect and the lack of statistical significance in the 3-month group may be indicative of difficulties in detecting training-associated improvements during the subacute recovery phase.

### 3.14 Subgroup for ischemic stroke

**Figure 11:**
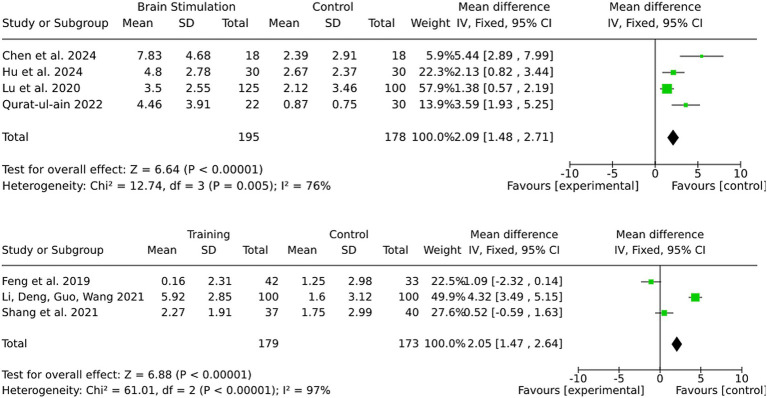
Subgroup for ischemic stroke.

Most studies in this review did not differentiate between stroke subtypes and included both ischemic and hemorrhagic stroke patients. However, a subset of studies exclusively investigated ischemic stroke patients, allowing for a separate analysis (Figure 11). For alternative medicine, none of the included studies differentiated between ischemic and hemorrhagic stroke patients, and no separate analysis was performed. For medication-based interventions, all included studies were conducted exclusively on ischemic stroke patients, ensuring that the observed effects are specific to this subgroup. For brain stimulation, the effect size in the ischemic stroke subgroup was 2.09 (95% CI: 1.48 to 2.71), closely matching the full-sample effect. Statistical significance remained strong (Z = 6.64, *p* < 0.00001), and heterogeneity was moderate (I^2^ = 76). For training interventions, the effect size in ischemic stroke patients was 2.05 (95% CI: 1.47 to 2.64), with very high heterogeneity (I^2^ = 97%). However statistical significance was notably stronger in ischemic stroke patients (*Z* = 6.88, *p* < 0.00001) compared to the mixed cohort, where results were not statistically significant.

## 4 Discussion

### 4.1 Summary of evidence

This systematic review was conducted to address the growing need for comprehensive, evidence-based insights into therapeutic interventions for PSCI. Unlike previous reviews that primarily focused on single intervention strategies, this review offers a comparative analysis of different therapeutic approaches to determine the most effective treatments for post-stroke cognitive recovery. The findings of the present systematic review indicate that brain stimulation therapies (particularly tDCS) and pharmacological interventions result in the most consistent and significant cognitive benefits.

Both brain stimulation methods, repetitive transcranial magnetic stimulation (rTMS) and transcranial direct current stimulation (tDCS), demonstrated significant and clinically meaningful effects. However, tDCS was found to show slightly greater and more consistent outcomes across studies. Interventions initiated earlier (within 3 months post-stroke) yielded more reliable effects. The results of the study indicated that the choice of comparator had a significant impact on the outcomes observed. Interestingly, when active brain stimulation was compared to no intervention, instead of sham stimulation, larger effects were found. This suggests that there may be a placebo effect associated with sham stimulation.

Pharmacological interventions exhibited the strongest and most consistent benefits, accompanied by low heterogeneity, suggesting the potential for generalization across diverse patient populations. Alternative medicine approaches, particularly acupuncture, showed significant benefits; however, the high heterogeneity of the results raises concerns about the consistency and reproducibility of these findings. Cognitive training was found to be effective and reliable in enhancing cognitive functions, whereas physical training demonstrated mixed outcomes with substantial variability, reflecting the need for further standardization and evaluation.

### 4.2 Mechanisms of action

#### 4.2.1 Brain stimulation

The mechanisms by which tDCS and rTMS promote recovery following a stroke are thought to involve the modulation of cortical excitability and the promotion of neuroplasticity (33). tDCS functions by applying a low electrical current to the scalp, which alters the resting membrane potential of neurons, thereby enhancing or inhibiting their excitability depending on the polarity of the stimulation (33). In contrast, rTMS involves the use of magnetic fields to generate electrical currents within the brain, thereby facilitating either excitation or inhibition of neuronal activity, depending on the stimulation frequency (34). The physiological mechanisms underlying these stimulation techniques involve modulation of neurotransmitter systems, particularly glutamate and gamma aminobutyric acid (GABA), which are essential for synaptic plasticity and cognitive recovery (35, 36). Research has demonstrated that both tDCS and rTMS can substantially enhance cognitive outcomes in stroke patients, particularly when employed in conjunction with cognitive rehabilitation strategies (37, 38). The timing of intervention has been demonstrated to exert a substantial influence on the efficacy of these brain stimulation techniques; studies have shown that initiating treatment within 3 months post-stroke yields more reliable outcomes in comparison to later interventions. This timeframe corresponds to a period of heightened neuroplasticity, which facilitates the reorganization of neural circuits implicated in cognitive functions (35, 39).

### 4.3 Pharmacological interventions

The studies investigating pharmacological interventions to improve cognitive function after stroke used either butylphthalide and oxiracetam (28) or ginkgo diterpene lactone meglumine (27). These compounds have neuroprotective properties and promote cognitive recovery through various mechanisms. Butylphthalide, a compound derived from the seeds of *Apium graveolens*, have been shown to exert significant neuroprotective effects in the context of ischemic stroke. Its mechanisms of action include the reduction of oxidative stress, inhibition of platelet aggregation, and modulation of inflammatory responses (28, 40). Specifically, butylphthalide has been reported to block ischemic damage through multiple pathways, thereby reducing the infarct area in animal models of stroke (28). Furthermore, it enhances cerebral blood flow and promotes neurogenesis, which are critical for cognitive recovery after a stroke (41). The combination of butylphthalide with oxiracetam, a nootropic agent known to enhance cholinergic function and improve cognitive performance, further enhances these effects. Oxiracetam increases the concentration of acetylcholine in the brain, which is essential for memory and learning processes (42). *Ginkgo Biloba* has long been used in herbal medicine, but recent research has focused on its active derivative, Ginkgo diterpene lactone meglumine (GDLM). GDLM is recognized for its neuroprotective properties, particularly in the treatment of ischemic stroke. It contains active components such as ginkgolides A, B, and K, which are known to exert antioxidant effects and protect against neuronal damage (43, 44). The pharmacological effects of GDLM include the modulation of inflammatory pathways and the inhibition of platelet-activating factor (PAF), which plays a critical role in the pathophysiology of stroke (45). By antagonizing PAF receptors, GDLM reduces inflammation and promotes neuroprotection, thereby facilitating cognitive recovery (46, 47). In addition, GDLM has been shown to improve blood flow in the brain, further supporting its role in cognitive rehabilitation after stroke (43).

### 4.4 Alternative medicine (acupuncture)

Although acupuncture is an established practice in traditional Chinese medicine, its continued investigation in stroke rehabilitation is driven by recent studies exploring its effects on neuroplasticity and cognitive recovery. Research suggests that acupuncture may improve blood flow in the brain, which is critical for the delivery of oxygen and nutrients necessary for neuronal recovery and cognitive function (48, 49). This improved perfusion may help mitigate the effects of ischemic damage and promote recovery in cognitive domains affected by stroke. Acupuncture also plays a role in promoting synaptic reconstruction and inhibiting neuronal apoptosis. Studies suggest that acupuncture can balance ion levels and facilitate the transmission and expression of neurotransmitters that are essential for cognitive processes (48, 49). Furthermore, acupuncture has also been associated with the suppression of inflammatory pathways and oxidative stress, which are known to contribute to cognitive decline after stroke. Animal studies have demonstrated that acupuncture can inhibit the activation of nuclear factor kappa B (NF-κB) and p53, both of which are involved in inflammatory responses and apoptosis (50). By mitigating these detrimental processes, acupuncture may help to preserve cognitive function and promote recovery.

### 4.5 Training approaches

Cognitive and physical training are often part of the standard rehabilitation in the treatment of stroke patients. Through repeated tasks, cognitive training is thought to stimulate the brain to reorganize and form new neural connections (51). Physical training, particularly aerobic exercise, has also been shown to have a positive effect on cognitive function in stroke patients. Aerobic physical exercise increases cerebral blood flow, which is essential for the delivery of oxygen and nutrients to the brain, thereby supporting cognitive health (51, 52). The mechanisms of action for both cognitive and physical training include the modulation of neurotransmitter systems, enhancement of neurotrophic factors, and reduction of inflammation. For example, physical exercise has been associated with increased levels of brain-derived neurotrophic factor (BDNF), which plays a critical role in neurogenesis and synaptic plasticity (53). Similarly, cognitive training can lead to changes in structural and functional connectivity of the brain, promoting efficient cognitive processing (51). Furthermore, both training modalities can help mitigate the effects of post-stroke inflammation, which is known to contribute to cognitive decline (53). We observed a stronger effect of training interventions in ischemic stroke patients, which may be attributed to differences in neuroplasticity and recovery mechanisms compared to hemorrhagic stroke patients. These factors could influence the responsiveness to structured cognitive and physical training programs.

## 5 Strengths and limitations

This systematic review has several strengths and limitations. We strictly followed well-defined inclusion and exclusion criteria and focused on therapies for cognitive impairment after stroke, including brain stimulation techniques (tDCS and rTMS), pharmacotherapeutics, training approaches, and alternative medicine. This ensured that our results included a wide range of traditional and novel interventions. We searched two high quality databases, PubMed and Embase, which are well suited to capturing clinical and experimental studies in this field. By including only studies that used the MoCA as a cognitive assessment tool, we ensured consistency and comparability of outcomes across studies. In addition, subgroup analyses allowed us to explore differences in effectiveness based on intervention type and study characteristics, adding depth and granularity to our findings. We maintained methodological rigor by assessing the risk of bias for all included studies.

However, several limitations must be acknowledged. First, a significant proportion of the included studies were conducted in China, which may limit the generalizability to Western countries. High heterogeneity between studies, including variability in intervention protocols, study designs, and follow-up periods, limits the generalizability and quantitative synthesis of the results. Second, most included studies did not distinguish between ischemic and hemorrhagic stroke patients in their analyses, despite potential differences in treatment response. Third, the literature research was limited to PubMed and Embase, excluding other databases. Furthermore, the omission of unpublished or non-English language studies may have introduced publication bias. Fourth, the focus on the MoCA, while ensuring consistency, may have excluded relevant studies using alternative cognitive measures. Additionally, the included studies did not consistently specify which version of the MoCA was used (e.g., standard MoCA, MoCA-Blind, or culturally adapted versions) or the exact timing of the assessment. Since test duration can vary due to differences in patient literacy, cognitive status, and testing conditions, this lack of reporting limits comparability across studies. Differences in methodological quality between studies, such as variations in randomization and blinding, may also affect the reliability of pooled results. Finally, the exclusion of chronic stroke populations limits the applicability of the results to long-term rehabilitation contexts.

### 5.1 Implications for practice

Novel therapeutic approaches introduced in the last 5 years, particularly brain stimulation methods such as tDCS and rTMS, have shown significant and clinically meaningful benefits in improving cognitive outcomes. Given the slightly larger and more consistent results associated with tDCS, clinicians may consider prioritizing this intervention when planning rehabilitation programs. However, since the handling of newer technical devices is challenging and experienced staff in this field is rare, the implementation of these therapies is demanding. Early initiation of therapies, within 3 months after stroke, appears to be crucial for achieving reliable effects, emphasizing the importance of timely intervention. Cognitive training also emerged as an effective and reliable method to improve cognitive functions and should be included into comprehensive rehabilitation plans. Pharmacological therapies showed the strongest benefits, suggesting that they remain an essential part of stroke rehabilitation, particularly for enhancing cognitive recovery. While alternative medicine, such as acupuncture, showed promising results, its high variability in outcomes warrants cautious implementation, and physical training may require more standardized protocols to optimize its effects. These findings support a multimodal and individualized approach to rehabilitation, using the strengths of each intervention to address the different needs of stroke survivors.

### 5.2 Implications for research

This review underscores the necessity for future research to advance the field of stroke rehabilitation, highlighting several pivotal areas for exploration. The encouraging outcomes observed for tDCS and rTMS call for further investigation to refine stimulation protocols, ascertain optimal dosages, and explore combinations with other therapeutic modalities. The observation that comparator types exerted a substantial influence on outcomes, with pronounced effects observed against no intervention as opposed to sham controls, signifies a pressing need for more rigorous examination of placebo effects in brain stimulation studies. Additionally, while pharmacological interventions demonstrated robust and consistent benefits, research should focus on long-term outcomes, adverse effect profiles, and interactions with other therapeutic modalities. The high heterogeneity observed in alternative medicine, particularly acupuncture, underscores the need for well-designed, standardized trials to improve the reproducibility and reliability of these findings. Finally, the equivocal outcomes associated with physical training suggest a need for further standardization and evaluation of its role in cognitive recovery, as well as exploration of its synergistic potential with cognitive and brain stimulation therapies. A combination of different novel therapy approaches could further enhance their effectiveness. Future studies should explore this aspect in more detail and examine the effects of these interventions beyond the acute and sub-acute phases to provide insights into their applicability for chronic stroke populations.

## 6 Conclusion

This review underscores the need for a multimodal approach to PSCI rehabilitation. Among the most effective interventions, brain stimulation (particularly tDCS) and pharmacological treatments show robust cognitive benefits, while cognitive training remains a cornerstone of rehabilitation. Physical training and alternative medicine approaches hold promise but require further standardization. Early intervention within the first 3 to 6 months is crucial for maximizing neuroplasticity, though later treatments can still lead to meaningful improvements.

Clinically, these findings support the integration of brain stimulation and pharmacological therapies into standard rehabilitation while emphasizing individualized and interdisciplinary treatment plans. Future research should refine stimulation protocols, assess long-term pharmacological effects, and explore the synergy of combined interventions. By shifting toward an integrated, network-based approach of PSCI, rehabilitation strategies can be optimized to better address the diverse needs of stroke survivors, ultimately enhancing long-term functional independence and quality of life.

## Data Availability

The original contributions presented in the study are included in the article/Supplementary material, further inquiries can be directed to the corresponding author.
