# Supplementary material for: Novel therapies for post-stroke cognitive impairment: a systematic review
Source: Front Neurol. 2025 May 27;16:1569329. doi: 10.3389/fneur.2025.1569329 (PMC12150855; doi:10.3389/fneur.2025.1569329)
Supplement: Supplementary file 2 [file Table_2.docx]

Table 2 Risk of Bias Evaluation; D1 Randomization process, D2 Deviations from intended interventions, D3 Missing outcome data, D4 Measurement of the outcome, D5 Selection of the reported results. Symbols: + = low risk of bias, - = some concerns, x = high risk of bias

|  | D1 | D2 | D3 | D4 | D5 | Overall |
| --- | --- | --- | --- | --- | --- | --- |
| Ai et al. 2024 | 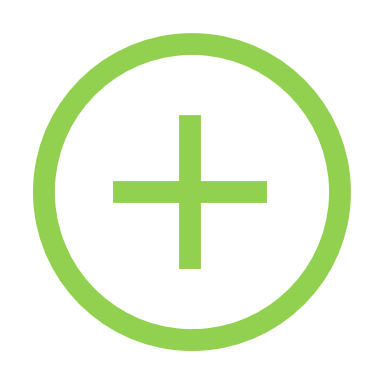 | 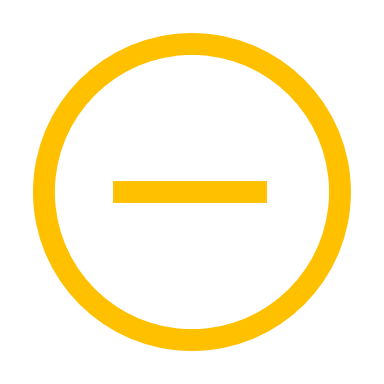 | 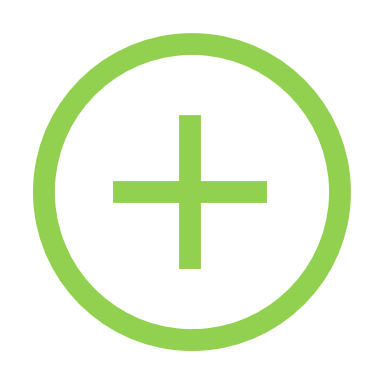 | 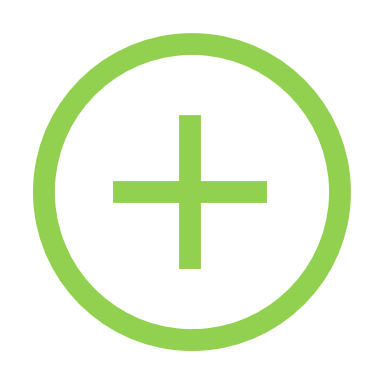 | 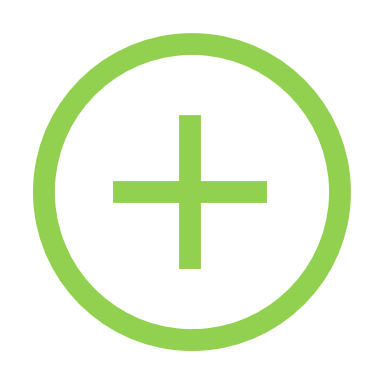 | 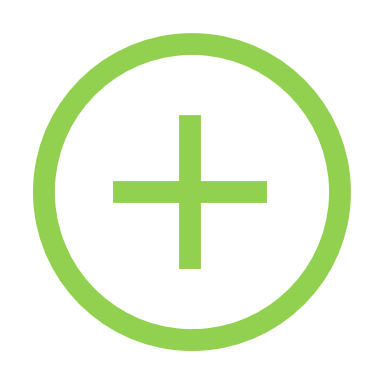 |
| Chen et al. 2020 | 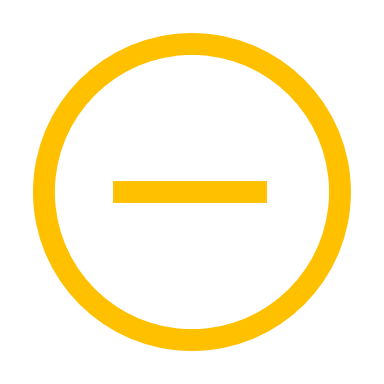 | 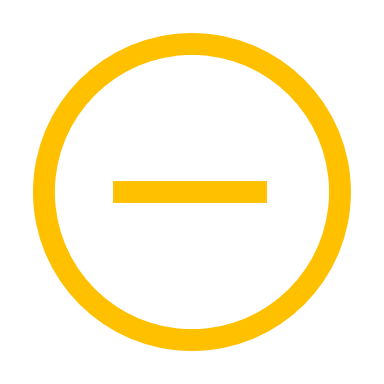 | 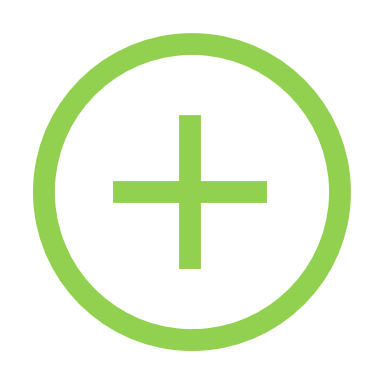 | 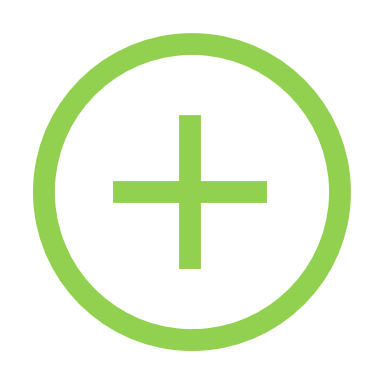 | 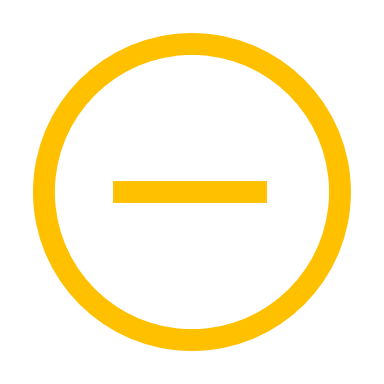 | 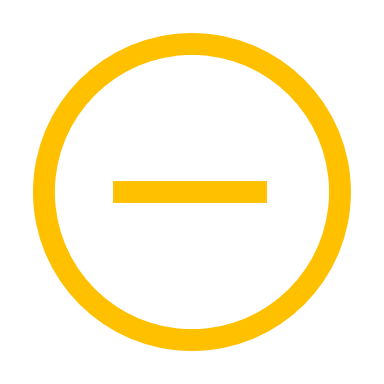 |
| Chen et al. 2024 | 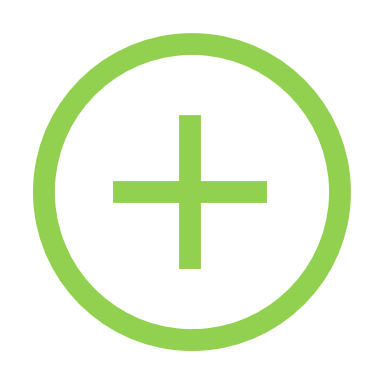 | 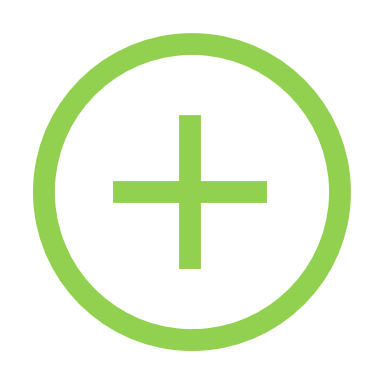 | 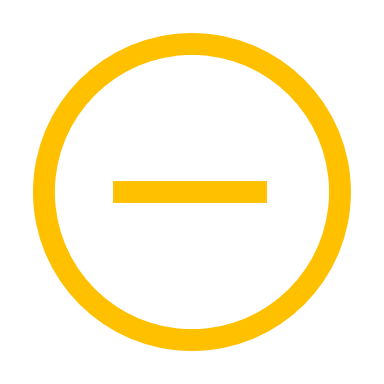 | 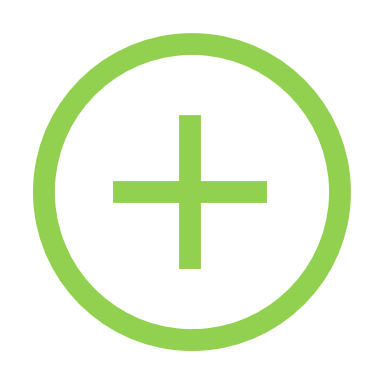 | 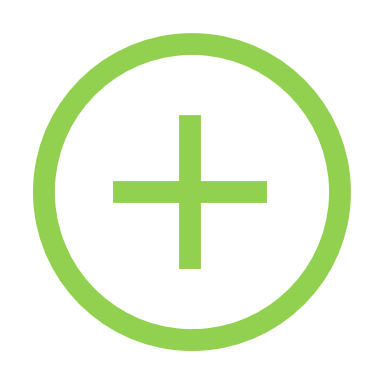 | 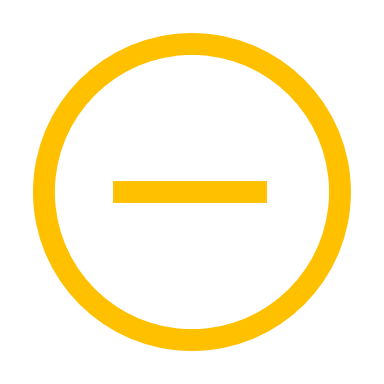 |
| Feng et al. 2019 | 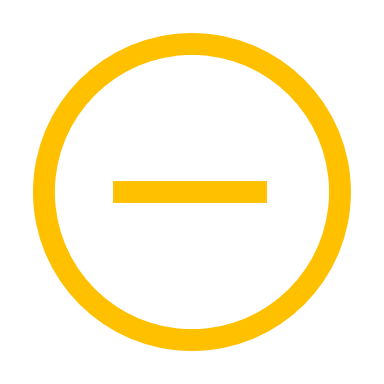 | 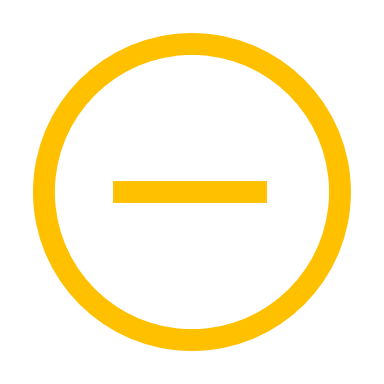 | 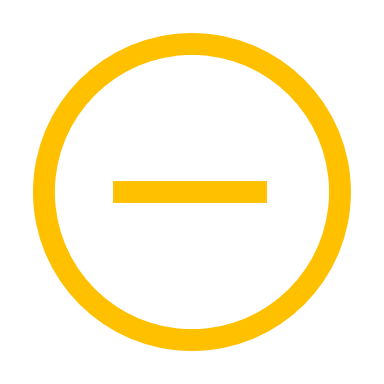 | 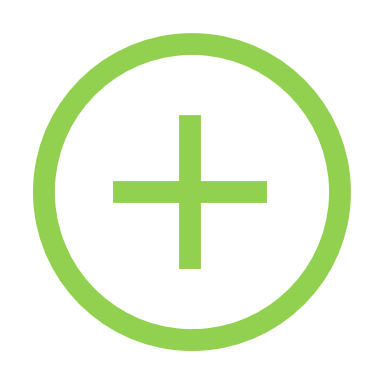 | 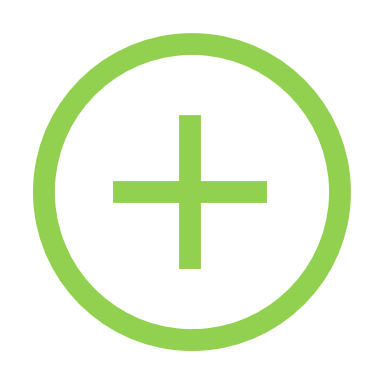 | 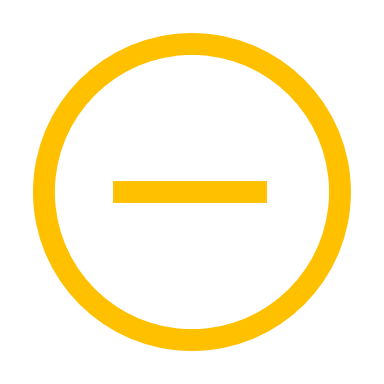 |
| He et al. 2021 | 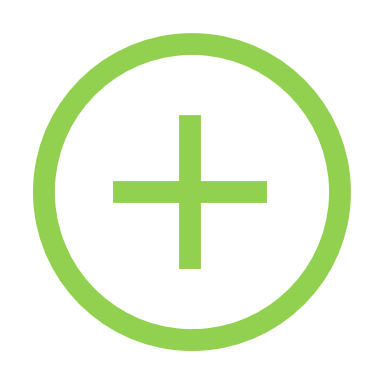 | 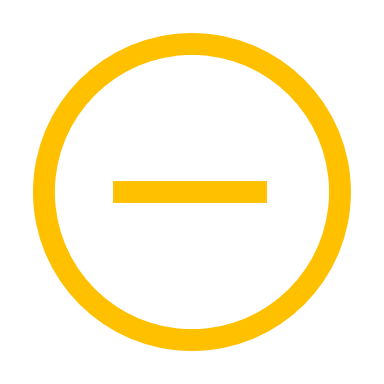 | 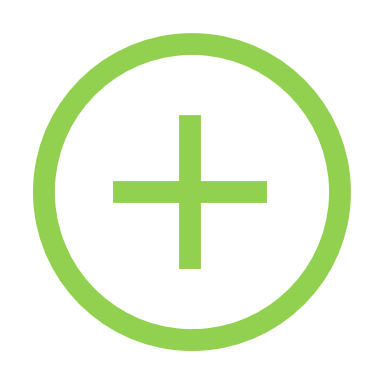 | 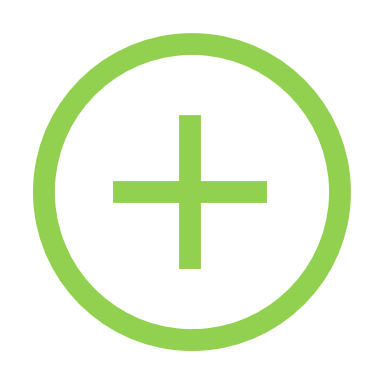 | 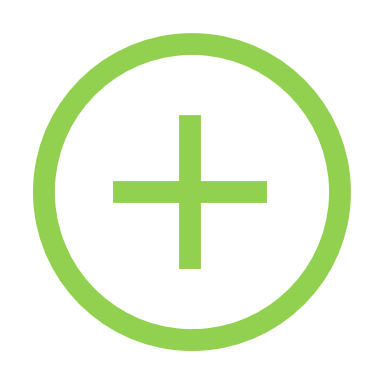 | 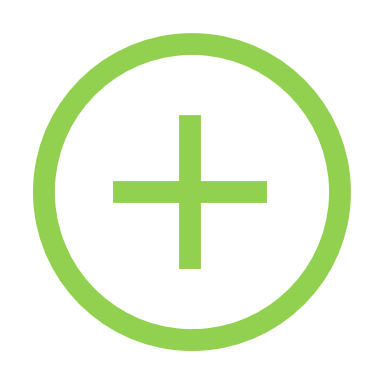 |
| Hu et al. 2024 | 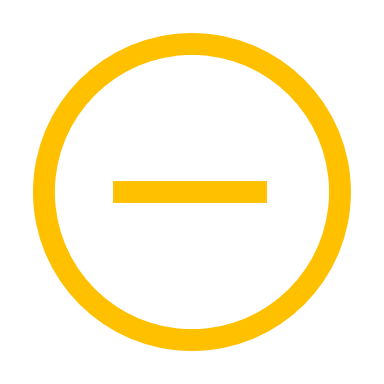 | 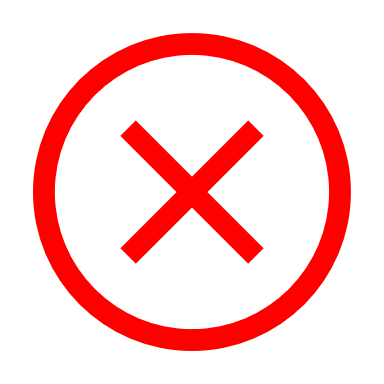 | 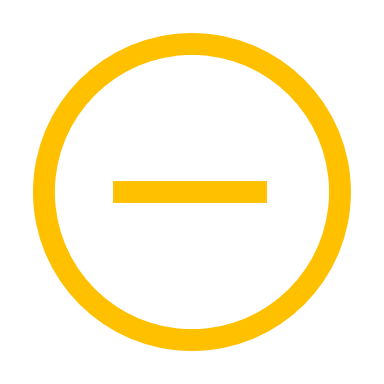 | 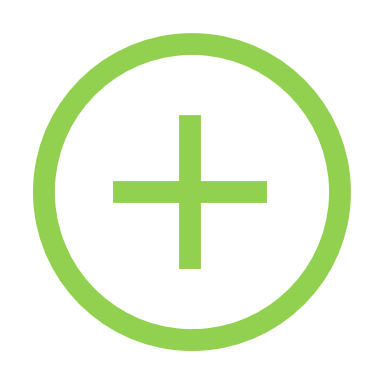 | 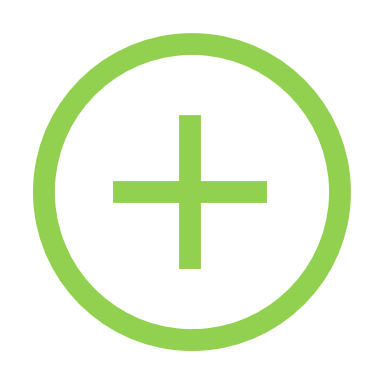 | 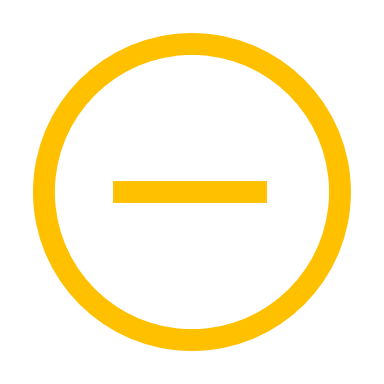 |
| Jiang, Yu & Deng 2023 | 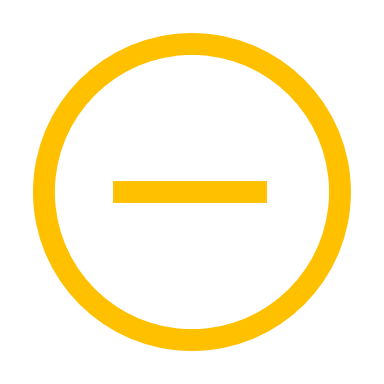 | 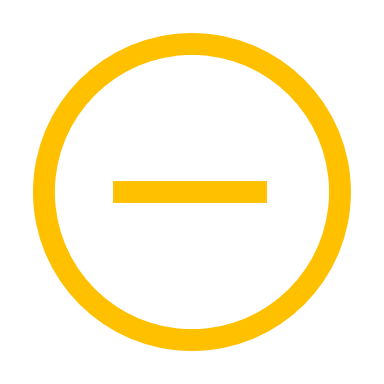 | 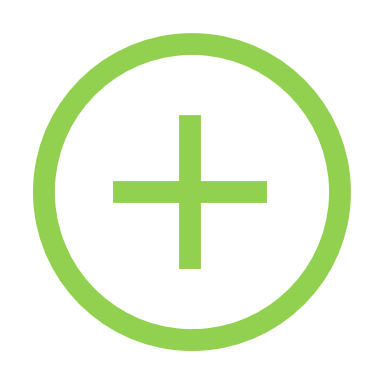 | 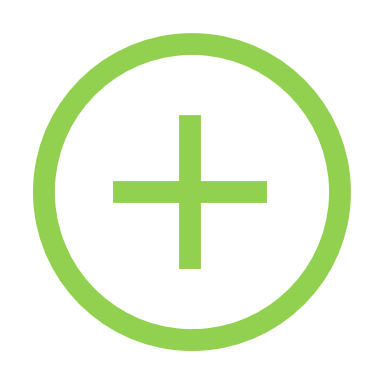 | 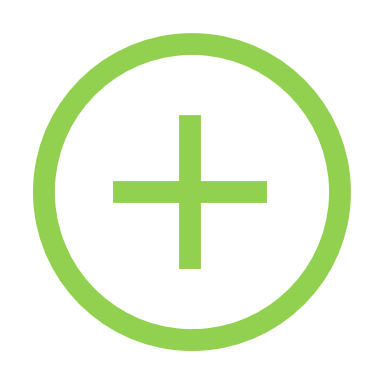 | 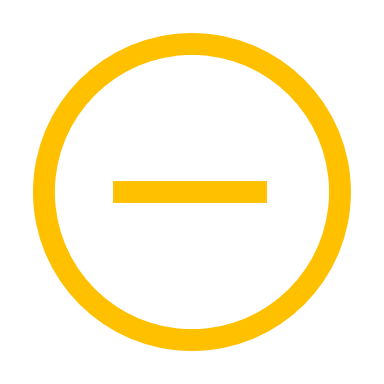 |
| Li et al. 2020 | 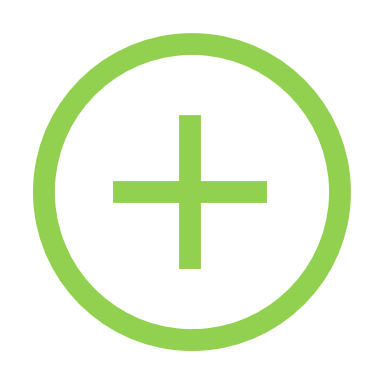 | 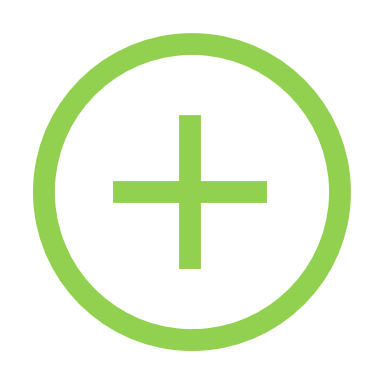 | 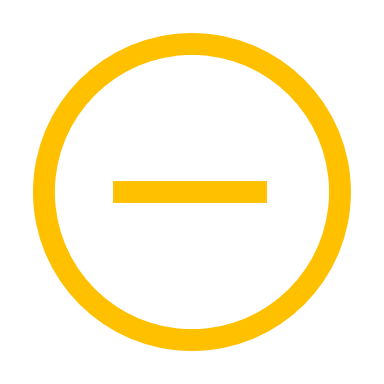 | 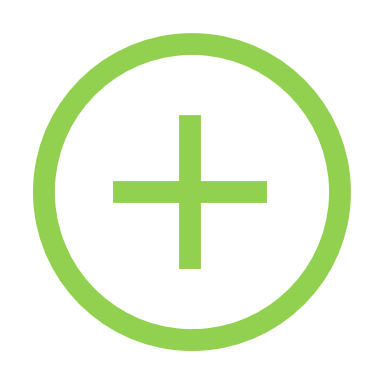 | 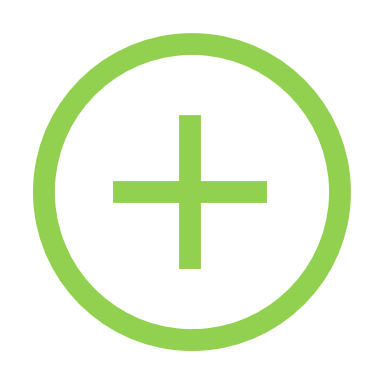 | 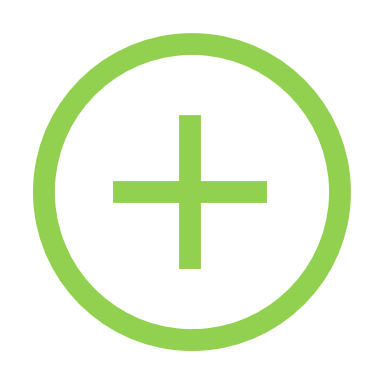 |
| Li et al. 2021 | 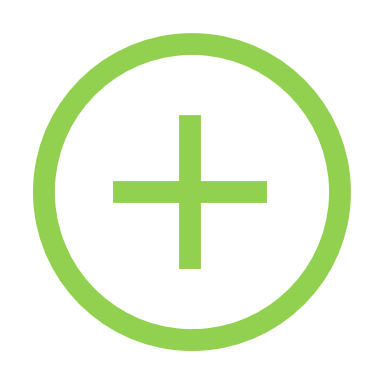 | 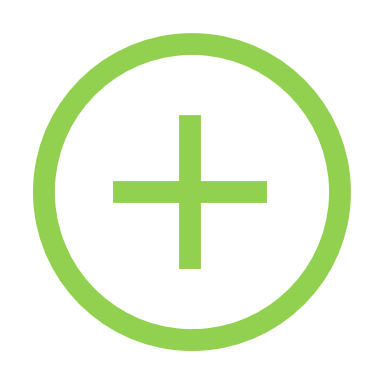 | 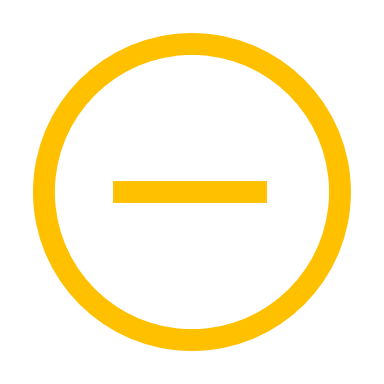 | 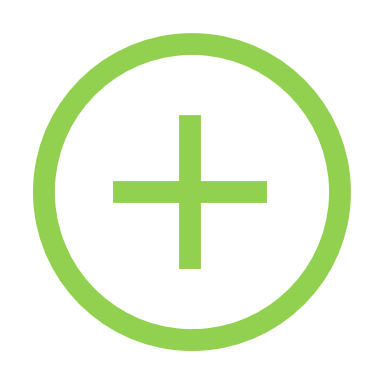 | 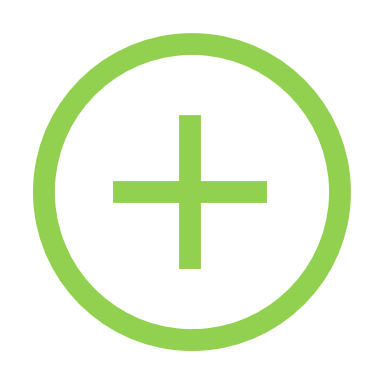 | 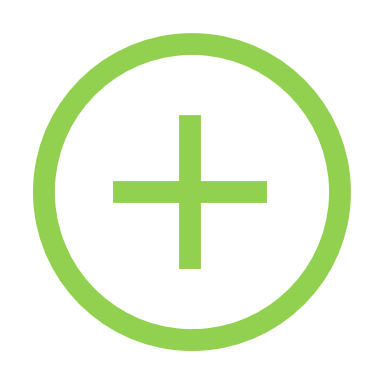 |
| Li, Deng, Guo & Wang 2021 | 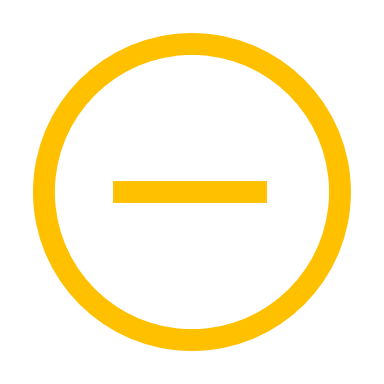 | 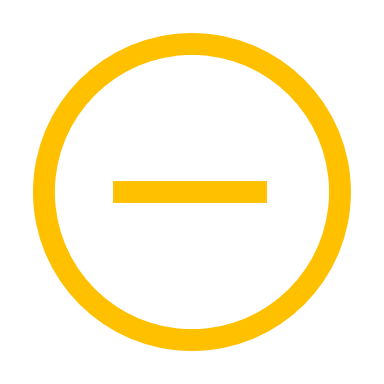 | 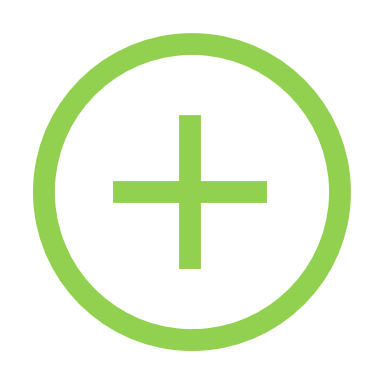 | 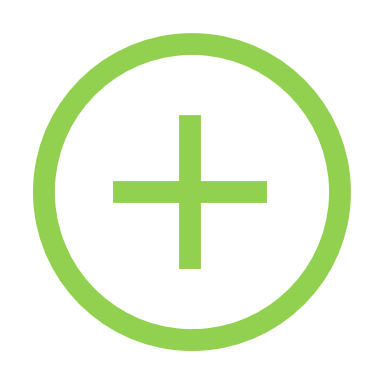 | 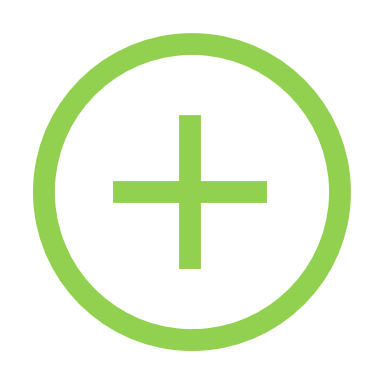 | 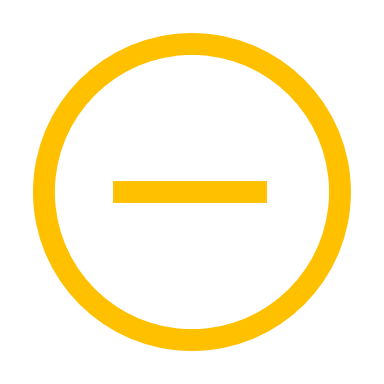 |
| Lu et al. 2020 | 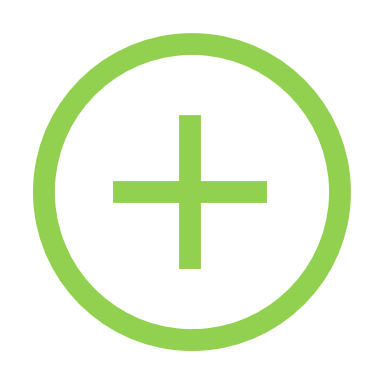 | 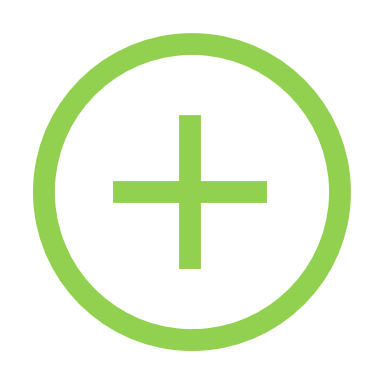 | 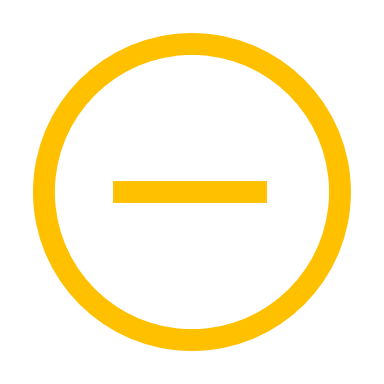 | 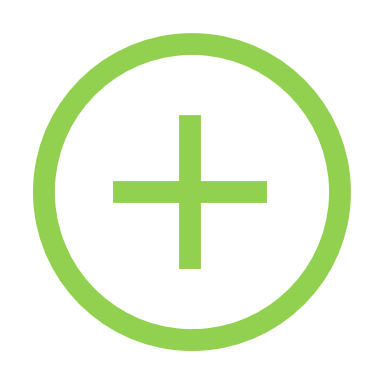 | 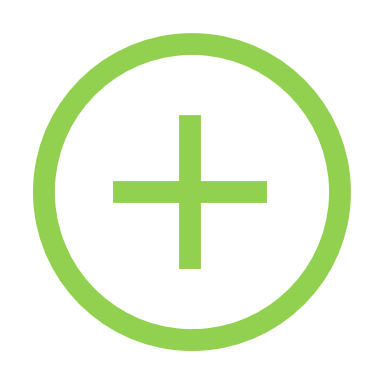 | 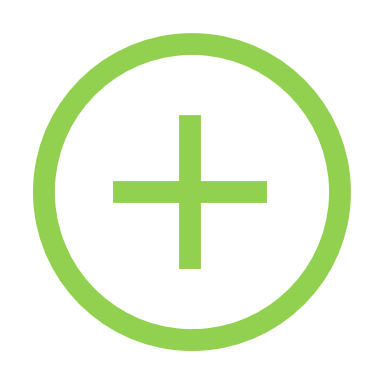 |
| Qurat-ul-ain et al. 2022 | 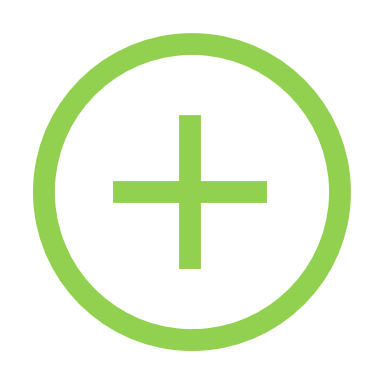 | 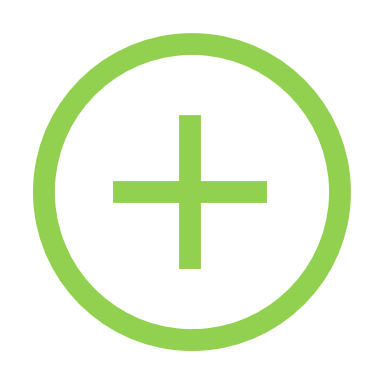 | 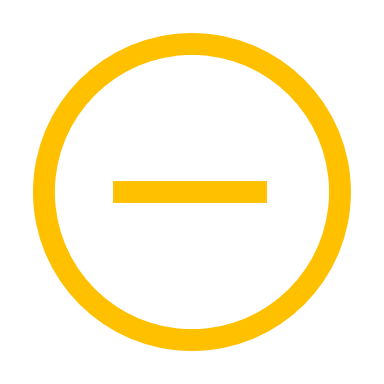 | 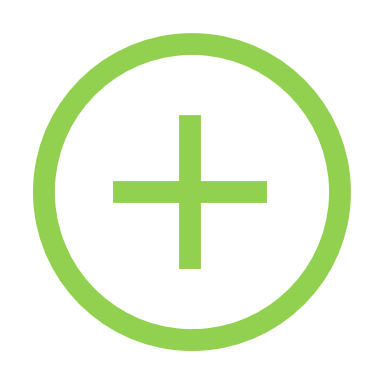 | 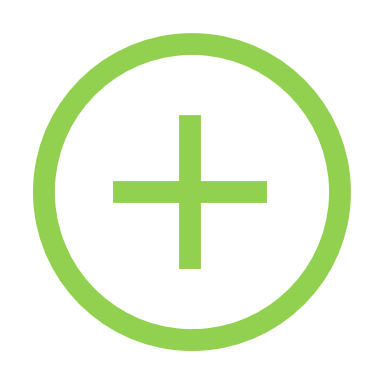 | 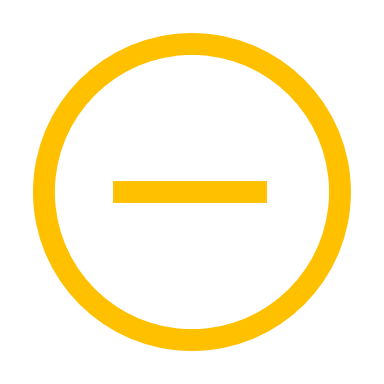 |
| Shang et al. 2021 | 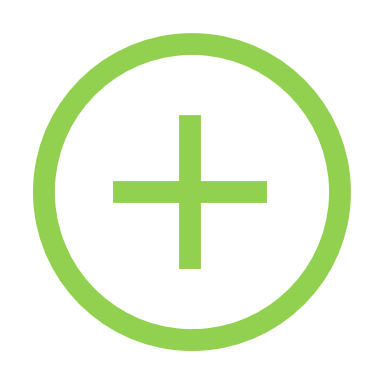 | 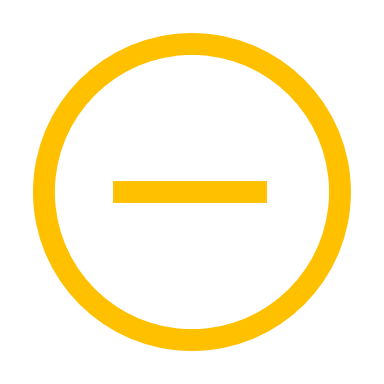 | 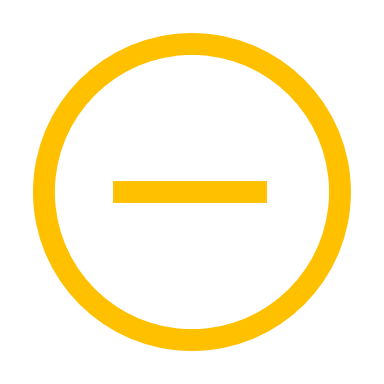 | 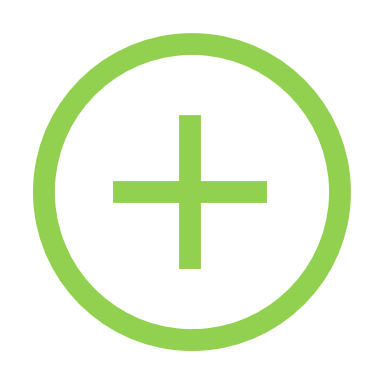 | 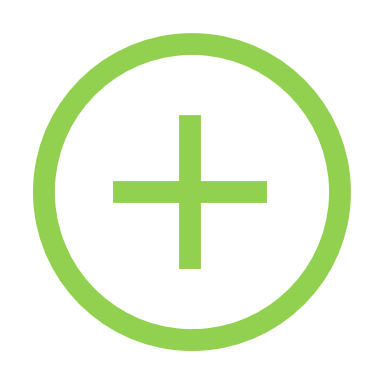 | 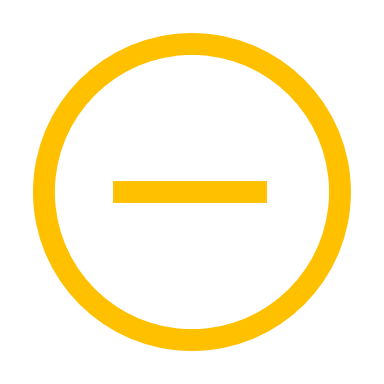 |
| Sun et al. 2022 | 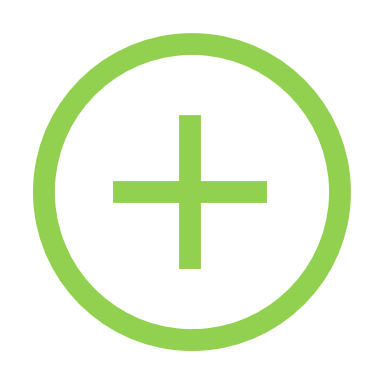 | 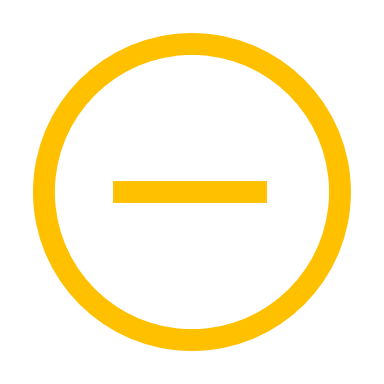 | 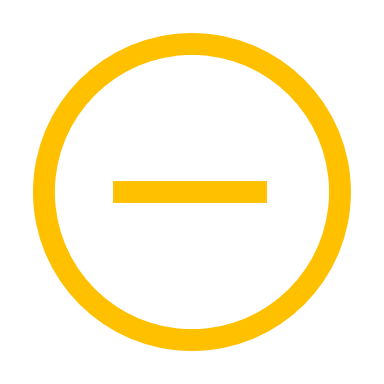 | 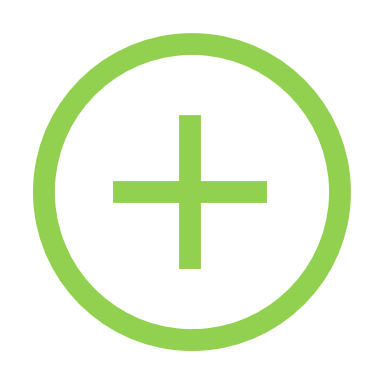 | 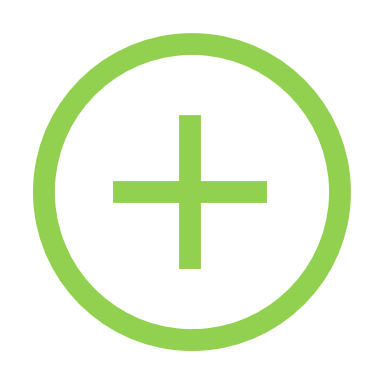 | 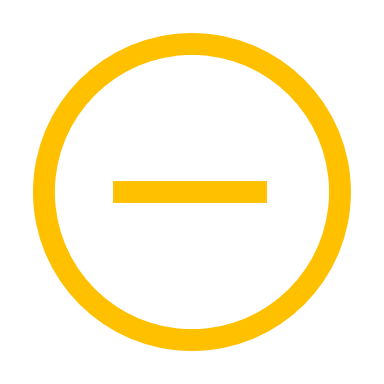 |
| Tian et al. 2024 | 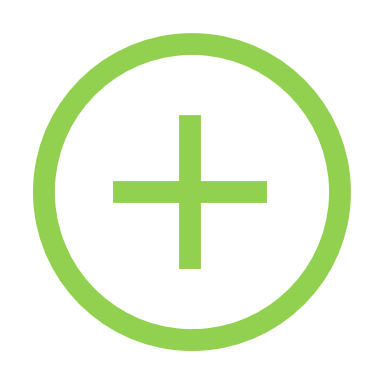 | 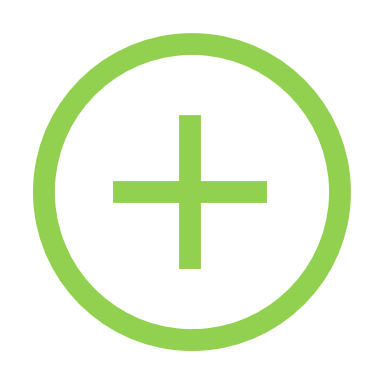 | 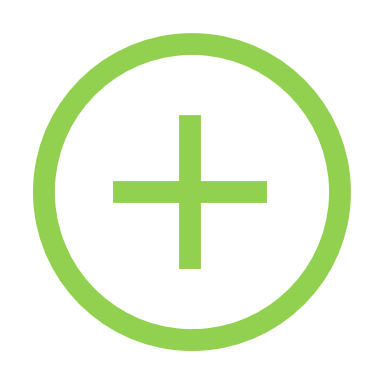 | 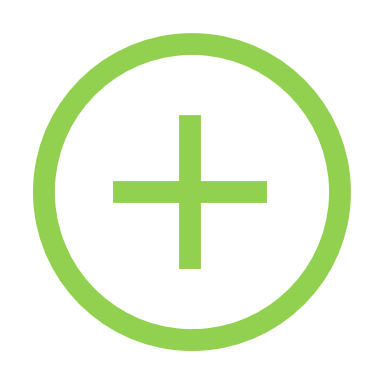 | 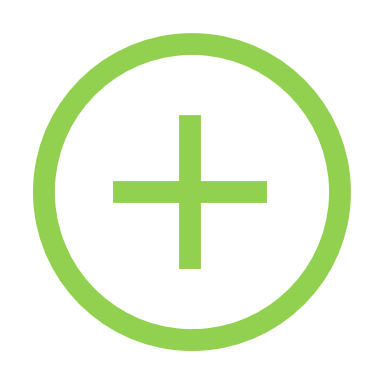 | 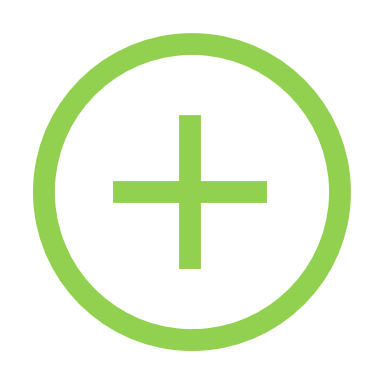 |
| Wang et al. 2021 | 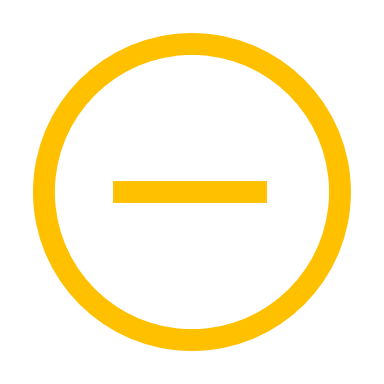 | 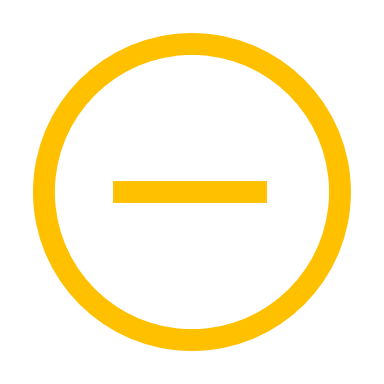 | 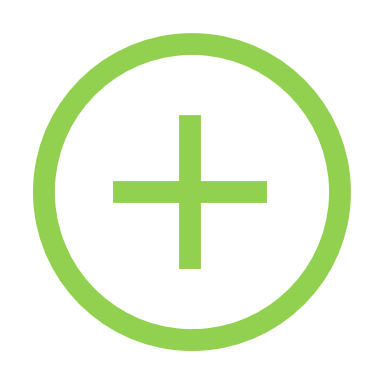 | 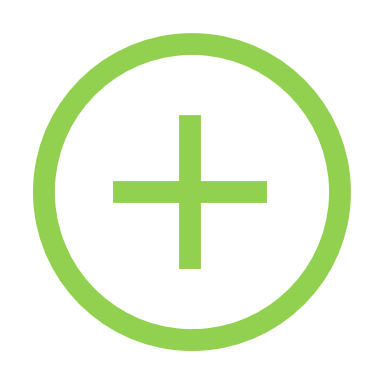 | 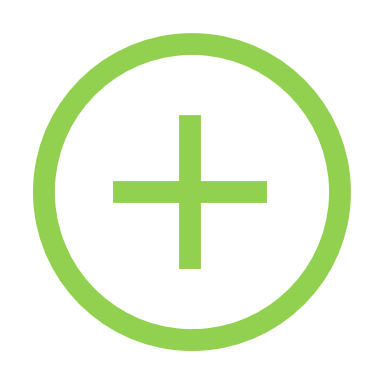 | 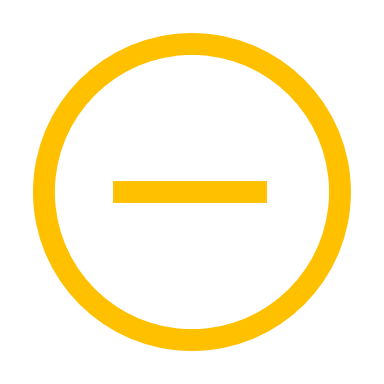 |
| Wilson et al. 2021 | 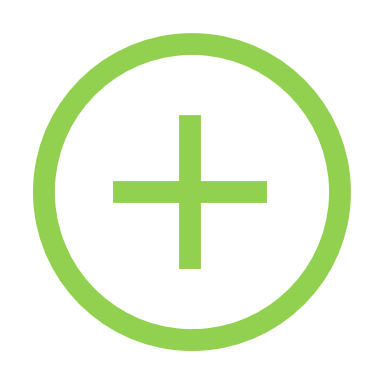 | 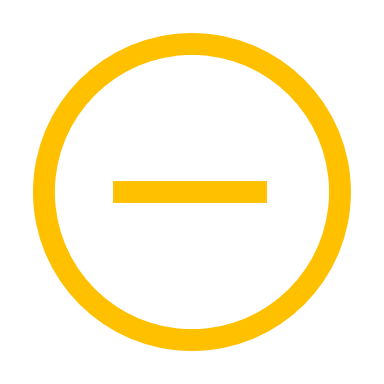 | 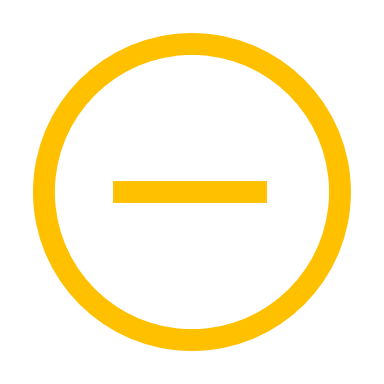 | 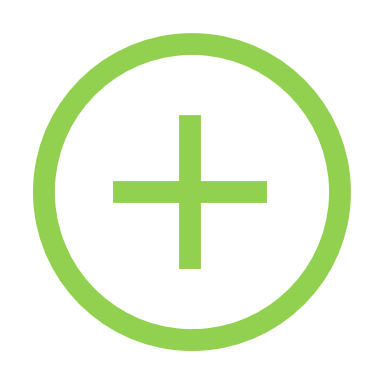 |  |  |
| Yang et al. 2022 |  |  |  |  |  |  |
| Yin et al. 2020 |  |  |  |  |  |  |
| Yu et al. 2024 |  |  |  |  |  |  |
| Zhang et al. 2022 |  |  |  |  |  |  |
| Zhang et al. 2024 |  |  |  |  |  |  |
